# Radiographers’ Recognition of Developmental Dysplasia of the Hip on Paediatric Pelvic Radiographs and Perceived Image‐Quality Factors: An Exploratory Observer‐Performance Study

**DOI:** 10.1155/rrp/8279959

**Published:** 2026-10-25

**Authors:** Niall Carr, Andrew England, Mark F. McEntee, Ben Treanor, Salman Mohammed Albeshan, Mohammadreza Elhaie

**Affiliations:** ^1^ University College Cork Cork Ireland ucc.ie; ^2^ Medical Imaging and Radiation Therapy University College Cork Cork Ireland ucc.ie; ^3^ Department of Radiological Sciences College of Applied Medical Sciences King Saud University Riyadh Saudi Arabia ksu.edu.sa; ^4^ Department of Radiology School of Medicine Isfahan University of Medical Sciences Riyadh Saudi Arabia mui.ac.ir

**Keywords:** abnormality recognition, developmental dysplasia of the hip (DDH), image quality, observer performance, paediatric radiography, preliminary image evaluation, radiographers

## Abstract

**Introduction:**

Developmental dysplasia of the hip (DDH) encompasses a spectrum of acetabular and hip‐joint abnormalities that may progress from dysplasia and instability to subluxation or frank dislocation. Radiographers are central to the acquisition of paediatric pelvic radiographs, but their ability to recognise radiographic features suggestive of DDH is not well established. This exploratory study evaluated abnormality recognition rather than formal diagnostic reporting and described radiographers’ confidence and perceived image‐quality requirements.

**Methods:**

A prospective, cross‐sectional observer‐performance study was conducted at the European Congress of Radiology 2023. Thirty radiographers independently reviewed 25 anonymised paediatric anteroposterior pelvic radiographs selected from the DDH teaching dataset. The observer set comprised 13 normal and 12 DDH‐positive radiographs: four left‐sided, four right‐sided and four bilateral cases. Reference labels were provided by three specialists in the source dataset. For each case, participants selected bilateral DDH, right‐sided DDH, left‐sided DDH, or no DDH; rated confidence from 1 to 10; and graded subjective image quality from perfect to inadequate. For the primary binary analysis, any response indicating DDH in at least one hip was classified as DDH‐positive. A unilateral response to a bilateral case was therefore counted as positive because it would prompt referral or further assessment. Observer characteristics and technical factors were described but were not tested as independent predictors of performance.

**Results:**

Mean reported sensitivity for DDH detection was 56.2% (SD 21.6%; range 0%–92.3%), and mean reported specificity was 40.2% (SD 28.4%; range 0%–100%). Confidence was generally moderate, with image‐level mean ratings predominantly between 4 and 6. Participants most frequently rated appropriate collimation, absence of pelvic rotation, lower‐limb positioning, radiographic centring and removal of artefacts as important technical considerations. These descriptive findings do not establish statistically significant predictors of observer performance.

**Conclusion:**

Radiographers demonstrated substantial variability and overall limited recognition of DDH in this small, convenience‐sampled observer study. The findings support further development and prospective evaluation of targeted education and clearly defined preliminary image‐evaluation pathways, but they do not establish that radiographer flagging, training, or artificial intelligence support improves diagnostic timeliness or patient outcomes.

## 1. Introduction

Developmental dysplasia of the hip (DDH) comprises a spectrum of developmental abnormalities involving acetabular morphology, hip instability, subluxation and dislocation. Delayed recognition may increase the need for invasive treatment and is associated with persistent pain, gait disturbance and premature osteoarthritis [1–8]. Epidemiological studies also demonstrate substantial geographic variation in prevalence and recognised perinatal risk factors [2, 3, 6, 7]. Imaging is age‐dependent: Ultrasonography is the principal modality in early infancy, whereas anteroposterior (AP) pelvic radiography becomes increasingly important as the femoral head ossifies and is commonly used from approximately 4 to 6 months of age and thereafter [9–11].

Interpretation of paediatric pelvic radiographs should therefore rely on age‐appropriate landmarks rather than adult‐only measures. The acetabular index and the relationship of the proximal femur to Hilgenreiner and Perkin lines are particularly important in younger children; continuity of Shenton’s line provides an additional sign of abnormal hip position, and Tönnis or International Hip Dysplasia Institute (IHDI) classifications may be used when grading displacement is appropriate. Centre‐edge measurements become more informative only after sufficient ossification and in older children [9, 11, 12]. These distinctions are important because the perceptual task may range from recognising obvious subluxation or dislocation to identifying more subtle acetabular dysplasia.

Radiographers have a primary responsibility for producing technically adequate examinations, but in some healthcare systems, they also undertake preliminary image evaluation or abnormality‐flagging processes analogous to the ‘red dot’ approach used in emergency radiography. In the present study, ‘recognition’ refers to a preliminary identification that an image may contain DDH‐related abnormality; it does not imply independent formal diagnosis or replacement of interpretation by radiologists, paediatricians or orthopaedic surgeons. In a future clinical pathway, a radiographer‐generated alert could be triggered by clear femoral‐head displacement/dislocation or by suspicious age‐appropriate acetabular and positional features, with the purpose of prompting timely review by the responsible interpreting clinician. Any decision to repeat a technically inadequate radiograph would remain governed by local paediatric radiation‐protection and repeat‐imaging protocols.

Despite the radiographer’s central role in image acquisition, evidence regarding radiographers’ recognition of DDH on paediatric pelvic radiographs remains limited. The present exploratory observer‐performance study therefore aimed to quantify radiographers’ binary recognition of DDH on a fixed image set and to describe diagnostic confidence, subjective image‐quality ratings and perceived technical requirements. Because no formal inferential analysis of observer characteristics or image‐quality variables was available, the study does not claim to identify independent factors statistically associated with performance.

## 2. Methods

### 2.1. Study Design

This exploratory, prospective cross‐sectional observer‐performance study used a structured electronic questionnaire administered through the European Federation of Radiographer Societies (EFRS) Research Hub during the European Congress of Radiology (ECR) 2023 in Vienna, Austria. Participation was voluntary. Ethical approval was obtained from the University College Cork Social Research Ethics Committee, and participants provided electronic informed consent after reviewing the participant information sheet. The study was designed to assess preliminary abnormality recognition rather than formal diagnostic reporting.

### 2.2. Case Set and Reference Standard

Twenty‐five anonymised paediatric AP pelvic radiographs were selected from the DDH X‐ray teaching dataset reported by Fraiwan et al. (Mendeley Data, Version 2) [13, 14]. The parent dataset contains 354 infant AP pelvic radiographs, including 120 labelled DDH cases and 234 normal cases, with a reported mean age of 4.5 ± 0.83 months and a maximum age of 7 months. The source images were acquired during standard diagnostic or screening examinations at King Abdullah University Hospital and Alsafa Specialist Hospital. The source publication reports that the reference labels were established by three specialists through radiographic inspection. The investigators conducting the present observer study did not independently readjudicate those source labels. The recovered reference key for the 25 observer‐study radiographs identified 13 normal and 12 DDH‐positive cases. The DDH‐positive cases comprised four left‐sided, four right‐sided and four bilateral cases, corresponding to a selected‐case DDH prevalence of 48%. Information on the sex, individual age, severity and previous treatment status of each selected case was not available. The source labels were treated as the operational reference standard. No formal sample‐size calculation was reported. The final study comprised 25 cases and 30 readers; this small fixed case set and convenience reader sample limit precision and generalisability.

### 2.3. Participants

Eligible participants were registered radiographers attending ECR 2023 who were willing to complete the observer study, including both clinically practising and academic radiographers. Previous paediatric imaging or DDH‐specific experience was not required for eligibility; instead, frequency of paediatric imaging, access to a dedicated DDH service, postgraduate DDH training, local protocol availability and years of clinical experience were recorded as participant characteristics. This clarification is consistent with the final sample, in which some participants reported no paediatric imaging activity. The sample was therefore a convenience sample and was not intended to be representative of all radiographers internationally. Participants who declined consent were excluded, and no personal identifiers were collected.

### 2.4. Observer Task and Scoring

For each of the 25 radiographs, participants answered three questions: (1) classification as bilateral DDH, right‐sided DDH, left‐sided DDH or no DDH; (2) confidence in that judgement on a 10‐point scale (1 = *no confidence*; 10 = *complete confidence*); and (3) subjective technical image‐quality rating using a four‐category scale (perfect, good, moderate or inadequate). The observer task was performed per radiograph rather than per individual hip. For calculation of binary sensitivity and specificity, the three responses indicating DDH in at least one hip were collapsed into a single DDH‐positive category, whereas ‘both hips normal’ was classified as DDH‐negative. Consequently, a response identifying only one affected hip in a bilateral DDH case remained positive in the binary analysis because the finding would prompt referral or further assessment. The binary analysis assessed recognition of the presence of DDH and did not measure laterality‐specific accuracy.

### 2.5. Image‐Quality and Technical‐Factor Assessment

Participants also rated the importance of predefined technical considerations, including artefact removal, pelvic rotation, lower‐limb positioning, lead‐shield placement, collimation, exposure‐factor selection, radiographic centring and side markers. These responses represent perceived technical requirements. The study did not include an independent expert image‐level technical‐quality adjudication linked to the reference standard, and no formal image‐level analysis was performed to test whether rotation, positioning, collimation, exposure, artefact or other technical deficiencies were associated with correct DDH recognition.

### 2.6. Data Collection

The questionnaire was developed and administered using Qualtrics software (Qualtrics, Seattle, WA, USA). Completed responses were stored on the Qualtrics platform and exported for analysis after study completion. The complete deidentified Qualtrics export, cleaned reader‐by‐case dataset, case reference key and original analysis files are retained securely within the research team. All 30 included participants completed the 25‐case observer task; no responses were recorded as missing, incomplete or excluded. Access to participant‐level records is restricted to the principal investigator and supervisory team from the Discipline of Medical Imaging and Radiation Therapy at University College Cork. The files are stored in a password‐protected, multifactor‐authenticated institutional project folder. See Supporting information file.

### 2.7. Data Analysis

Data were transferred from Qualtrics to Microsoft Excel (Microsoft, Redmond, WA, USA) for processing and descriptive analysis. Sensitivity and specificity were calculated for each participant at the radiograph level after the four response categories were collapsed into binary DDH‐positive or DDH‐negative classifications as described above. Participant‐level sensitivity and specificity were summarised using the mean, standard deviation (SD), minimum and maximum. Demographic characteristics, confidence ratings, subjective image‐quality ratings and perceived technical requirements were summarised descriptively. No formal inferential comparisons were performed between observer characteristics and sensitivity or specificity, and the study therefore does not identify independent predictors of performance. No multireader, multicase model, mixed‐effects analysis, interobserver‐agreement statistic or image‐quality‐by‐accuracy analysis was performed. The original deidentified reader‐level data and analysis materials are retained securely by the research team and remain available for internal verification. An exploratory receiver operating characteristic (ROC) display was generated during the original analysis. Because the analysis did not adjust for the crossed reader‐by‐case structure, the reported AUC is presented descriptively and is not used to support inferential conclusions.

## 3. Results

Thirty radiographers completed the questionnaire during ECR 2023. Participant characteristics are summarised in Table 1. The sample was internationally diverse, with 70% of participants from continental Europe. Experience varied widely, and the most common category was 1–5 years in practice (27%). Ten participants (33%) reported never performing paediatric imaging, whereas three (10%) reported daily paediatric imaging. Six participants (20%) reported access to a dedicated DDH service, three (10%) had postgraduate DDH training and six (20%) reported a specific local DDH protocol. These variables are reported descriptively and were not tested as predictors of observer performance.

**TABLE 1:** Participant demographics (*N* = 30).

Variable	*n* (%)
Country	
United Kingdom and Northern Ireland	5 (17)
Rest of Europe	21 (70)
Outside Europe	4 (13)
Clinical experience (years)	
< 1	4 (13)
1–5	8 (27)
6–10	3 (10)
11–15	7 (23)
16–20	4 (13)
> 20	4 (13)
Frequency of paediatric imaging	
Daily	3 (10)
Weekly	7 (23)
Monthly	5 (17)
Half‐yearly	2 (7)
Yearly	3 (10)
Never	10 (33)
Dedicated DDH service	
Yes	6 (20)
No	24 (80)
Postgraduate DDH training	
Yes	3 (10)
No	27 (90)
Specific local DDH protocol	
Yes	6 (20)
No	24 (80)

Participants rated several technical considerations as important for DDH image assessment. The highest reported percentages were for collimation (89.5%), absence of pelvic rotation (89.1%), lower‐limb positioning (87.6%), inclusion of side markers (85.7%), radiographic centring (85.5%) and removal of artefacts (81.0%). Lead‐shield placement and exposure‐factor selection were each reported at 66.8% (Table 2). These values represent participant‐reported perceptions and should not be interpreted as demonstrated determinants of diagnostic accuracy.

**TABLE 2:** Participant‐rated technical factors considered important for DDH image assessment.

Factor	Reported importance (%)
Removal of artefacts	81.0
Absence of patient rotation	89.1
Position of the lower limbs	87.6
Position of lead shielding	66.8
Collimation	89.5
Correct exposure‐factor selection	66.8
Correct radiographic centring	85.5
Inclusion of aspect/side markers	85.7

*Note:* Percentages are aggregated from the original survey responses. The complete deidentified response export and reader‐level ratings are retained securely by the research team; only aggregated results are presented here to protect participant confidentiality and remain within the scope of the original ethical approval.

Participant‐level DDH detection performance varied substantially. Mean sensitivity was 56.2% (SD 21.6%; range 0%–92.3%), and mean specificity was 40.2% (SD 28.4%; range 0%–100%) (Table 3). These measures represent binary recognition of DDH presence at the radiograph level. They do not measure whether the participant identified the correct affected side or distinguished unilateral from bilateral disease. The original deidentified reader‐by‐case dataset, case reference key and analysis files are retained securely by the research team.

**TABLE 3:** Summary of reported participant‐level DDH detection performance.

Metric	Mean (%)	SD (%)	Minimum (%)	Maximum (%)
Sensitivity	56.2	21.6	0	92.3
Specificity	40.2	28.4	0	100

*Note:* Sensitivity and specificity were calculated at the radiograph level after bilateral, right‐sided and left‐sided DDH responses were collapsed into a single DDH‐positive category. ‘Both hips normal’ was treated as DDH‐negative. A unilateral response to a bilateral DDH case was therefore positive for the binary analysis. Laterality‐specific accuracy, PPV and NPV were not prespecified outcomes and are not reported.

Figure 1 displays the mean participant confidence rating for each image together with the upper and lower limits labelled as 95% confidence intervals in the original analysis. Image‐level mean confidence was generally moderate, predominantly between 4 and 6 on the 10‐point scale. Confidence ratings reflect certainty in each participant’s own classification and should not be interpreted as direct measures of DDH presence, technical quality or diagnostic correctness. The original reader‐level confidence ratings and analysis files are retained securely by the research team; aggregated values are presented to protect participant confidentiality.

**FIGURE 1:**
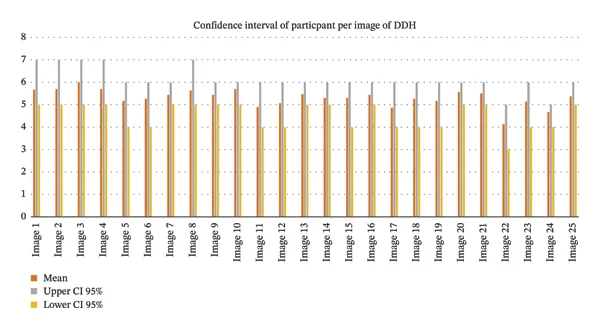
Mean participant confidence rating for each paediatric pelvic radiograph on a 10‐point scale (1 = *no confidence*; 10 = *complete confidence*), with the upper and lower limits labelled as 95% confidence intervals in the original analysis. The plotted values represent confidence in each participant’s classification, not the probability that DDH is present. Aggregated results are shown to protect participant confidentiality.

Figure 2 shows the exploratory ROC display generated during the original study analysis, for which an AUC of 0.6522 (SD 0.0307) was reported. Nondiagnostic trend lines were removed from the revised display. The original deidentified data and analysis files are retained securely within the research team. Because the analysis did not account for the crossed reader‐by‐case structure, the AUC is retained only as an exploratory descriptive result and is not treated as a primary inferential estimate.

**FIGURE 2:**
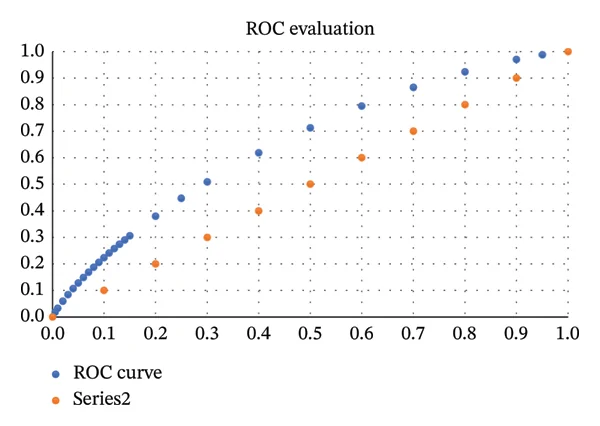
Exploratory receiver operating characteristic (ROC) display from the original study analysis. The reported AUC was 0.6522 (SD 0.0307). Nondiagnostic trend lines were removed in revision. Because the analysis did not account for reader‐by‐case clustering, the display is descriptive and is not used as a primary inferential result.

## 4. Discussion

### 4.1. Principal Findings

This exploratory observer‐performance study found wide variation and overall limited recognition of DDH among 30 radiographers reviewing 25 paediatric AP pelvic radiographs. Mean sensitivity was 56.2%, and mean specificity was 40.2%. Confidence was generally moderate. Participants frequently rated correct collimation, absence of rotation, appropriate lower‐limb positioning, radiographic centring and artefact removal as important; however, these were perceived technical requirements rather than statistically demonstrated predictors of correct interpretation. The study therefore supports a more cautious conclusion than the original wording: It describes baseline recognition performance and perceptions within a small convenience sample, but it does not establish causal or independent factors associated with performance.

### 4.2. Clinical Target and Proposed Workflow

Any future radiographer role should be framed as preliminary image evaluation rather than definitive DDH diagnosis. A practical alert threshold would need to be explicitly defined and taught. It could include obvious subluxation or dislocation and, where radiographers are trained to assess them, suspicious age‐appropriate features such as an abnormal acetabular index, abnormal relationships to Hilgenreiner or Perkin lines, disruption of Shenton’s line or displacement patterns consistent with Tönnis or IHDI categories [9, 11, 12]. A radiographer‐generated alert would then prompt timely review by the responsible radiologist or referring clinical team; it would not replace specialist interpretation. Likewise, technically inadequate imaging should trigger action only in accordance with local repeat‐imaging and radiation‐protection policies. The present study did not test such an escalation pathway, so any benefit for time to diagnosis remains hypothetical and requires prospective evaluation.

### 4.3. Image Quality, Experience and Training

The technical‐factor results should be interpreted as perceptions. No independent expert assessment established whether the 25 images contained rotation, tilt, positioning error, artefact, exposure error or suboptimal collimation, and no image‐level model linked technical quality to correctness. Similarly, years of experience, frequency of paediatric imaging, DDH‐specific training, dedicated‐service exposure and local protocol availability were recorded but were not formally tested against sensitivity or specificity. Accordingly, the present data do not establish whether regular paediatric imaging exposure is associated with better DDH recognition. Lead‐shield placement also warrants particular caution: Although some participants considered it important, an incorrectly positioned shield can obscure relevant anatomy or interfere with image acquisition. Its survey rating should not be interpreted as a recommendation for universal shielding practice.

### 4.4. Comparison With Automated Imaging Research

Artificial intelligence studies have reported strong performance for automated DDH assessment on radiographs and ultrasound [4, 13, 15–19], and MRI remains useful in selected complex or postreduction assessments [10, 20, 21]. Nevertheless, direct numerical comparison with the present observer results is inappropriate because AI studies may differ in case mix, prevalence, reference standards, image selection, outcome definitions, training/test separation and external validation. The current data therefore do not demonstrate that AI outperforms radiographers under matched conditions, nor that AI‐assisted radiographer review would reduce delayed diagnosis or improve clinical outcomes. Prospective studies using the same cases, reference standard and clinical workflow for human and AI assessment would be required before such claims could be made.

### 4.5. Broader Orthopaedic Context

DDH is a developmental acetabular disorder and should be distinguished from traumatic acetabular pathology, for which the morphology and integrity of the acetabulum influence reconstructive decision‐making in a different clinical context [22]. Congenital musculoskeletal pathology more broadly can also provide insight into prenatal mechanisms, although reports such as congenital posteromedial bowing of the tibia and fibula concern a distinct disorder and should not be extrapolated to DDH [23]. Looking ahead, any AI‐enabled orthopaedic imaging pathway will also require secure data provenance, governance and traceability; emerging digital approaches such as blockchain have been proposed for orthopaedic practice, although their clinical utility remains to be established [24].

### 4.6. Strengths and Limitations

Strengths include the international recruitment setting, a common image set assessed by every observer, an exact case reference key and collection of confidence and technical‐quality perceptions alongside DDH recognition. Several limitations remain substantial. Recruitment at a single international congress produced a small convenience sample with heterogeneous professional backgrounds and limits generalisability; one‐third of participants reported no paediatric imaging activity. Only 25 cases were used, and no formal sample‐size calculation was performed. The recovered case key establishes that the observer set contained 13 normal cases and 12 DDH‐positive cases, comprising four left‐sided, four right‐sided and four bilateral cases. However, individual case data on sex, age, DDH severity, previous treatment and objective technical quality were not available. The selected‐case prevalence and severity spectrum should not be assumed to represent routine clinical practice. The analysis was primarily descriptive; no multireader, multicase or mixed‐effects model, clustered confidence intervals, formal interobserver‐agreement statistic, observer‐factor analysis or image‐quality‐by‐accuracy analysis was performed. Because sensitivity and specificity were defined for binary DDH recognition, they do not describe laterality‐specific accuracy or distinguish partial from complete identification of bilateral disease. Finally, the exploratory ROC analysis did not adjust for reader and case clustering. These limitations mean that the reported sensitivity and specificity should be viewed as exploratory estimates rather than definitive measures of radiographer diagnostic competence.

Future research should use a prospectively defined, clinically representative case set with transparent age distribution, disease prevalence, laterality and severity strata and a clearly documented expert reference standard. Scoring rules should be prespecified for both binary recognition and laterality‐specific performance, and multireader, multicase or mixed‐effects methods should account for reader and case clustering. Future analyses should also assess interobserver agreement and examine how experience, training, objective image quality and local workflows relate to recognition performance. Representative normal, subtle dysplasia, subluxation and dislocation examples would improve reproducibility and educational value. If preliminary radiographer alerting or AI support is evaluated, studies should predefine the alert threshold and escalation pathway and measure clinically meaningful outcomes such as reporting time, referral delay, unnecessary repeat imaging and patient outcomes.

## 5. Conclusion

This exploratory study demonstrates substantial variability and overall limited DDH recognition among a small convenience sample of radiographers. The findings justify further work on age‐appropriate DDH education, standardised image‐acquisition principles and clearly defined preliminary image‐evaluation pathways. They do not establish that radiographer flagging, targeted training or AI decision support improves diagnostic timeliness or patient outcomes. Larger multireader, multicase studies with transparent reference standards, clinically representative case mixes, objective image‐quality assessment and prospectively defined escalation pathways are required before changes to routine clinical practice can be recommended.

## Funding

No funding was received for this manuscript.

## Conflicts of Interest

The authors declare no conflicts of interest.

## Supporting Information

Additional supporting information can be found online in the Supporting Information section.

## Supporting information


**Supporting Information** The complete deidentified Qualtrics export, cleaned reader‐by‐case dataset, case reference key and original analysis files are retained securely by the research team.

## Data Availability

The complete deidentified Qualtrics export, cleaned reader‐by‐case dataset, case reference key and original analysis files are retained securely by the research team. Participant‐level data are not publicly available because external disclosure was not included within the scope of the original ethical approval. The questionnaire, scoring description, case reference key and aggregated results may be provided where appropriate. Any confidential access to deidentified participant‐level data would require prior institutional ethics and data‐governance approval.
