# Supplementary material for: Radiographers’ Recognition of Developmental Dysplasia of the Hip on Paediatric Pelvic Radiographs and Perceived Image‐Quality Factors: An Exploratory Observer‐Performance Study
Source: Radiol Res Pract. 2026 Oct 25;2026:8279959. doi: 10.1155/rrp/8279959 (PMC13696998; doi:10.1155/rrp/8279959)
Supplement: Supplementary file 1 [file RRP-2026-8279959-s001.pdf]

Survey Questions

Q1 Please confirm where you are completing this data collection.

- ◯ EFRS Research Hub Vienna (2)
- ◯ Online (7)

Q2 In which country do you currently reside?

▼ Afghanistan (1) ... Zimbabwe (1357)

Q3 How many **years** have you been qualified as a radiographer?

- ◯ <1 year (1)
- ◯ 1-5 years (2)
- ◯ 6-10 years (3)
- ◯ 11-15 years (4)
- ◯ 16-20 years (5)
- ◯ >20 years (6)

Q4 In your current clinical practice, how often do you undertake or review paediatric pelvis X-ray images?

- ◯ Daily (1)
- ◯ Weekly (2)
- ◯ Monthly (3)
- ◯ Half Yearly (4)
- ◯ Yearly (5)
- ◯ Never (6)

Q5 Does the hospital / healthcare institution where you work provide a **dedicated service** for children with **suspected** or **known developmental dysplasia of the hip (DDH)**?

- ◯ Yes (1)
- ◯ No (2)
- ◯ No but there are plans to establish such a service (3)

Q6 Have you received any **post-qualification (registration) training** in the imaging of **developmental dysplasia of the hip (DDH)**?

- ◯ No (1)
- ◯ Yes - attended formal training outside of my institution (2)
- ◯ Yes - received in-house (via my employer) training (3)

- ◯ Other (4)

Q7 Do you have a **specific developmental dysplasia of the hip X-ray protocol** (beyond a standard paediatric pelvis protocol) in your institution / workplace?

- Yes (1)
- No (2)
- Don't know (3)

Q8 With respect to radiographic technique for imaging suspected developmental dysplasia of the hips (DDH), please rank which radiographic steps are the most important via this scale (10-30% not always essential, 30-70% generally important, 70-100% crucial to answer clinical question).

	0 10 20 30 40 50 60 70 80 90 100
Remove of artefacts (e.g. nappies / clothing) (1)	
Absence of patient rotation (2)	
Position of the lower limbs (3)	
Position of lead shielding (4)	
Collimation, inclusion of all relevant anatomy (5)	
Correct exposure factor selection (6)	
Correct radiographic centring (7)	
Inclusion of aspect / side markers (8)	

Page Break

End of Block: Block 2

Start of Block: Image 1 (N5)

Q9 (**Image 1**) You are now asked to consider this image of a child with suspected Development Dysplasia of the Hip (DDH). You will be asked to confirm whether this image is **normal** or **abnormal** (**for DDH only**) and indicate a **confidence rating** for your decision (0-not confident, 10-totally confident). You will also be invited to give **your opinion about the image quality**, this should consider ALL elements of image quality BUT NOT those which are exposure factor related (i.e. contrast, density, sharpness, noise).

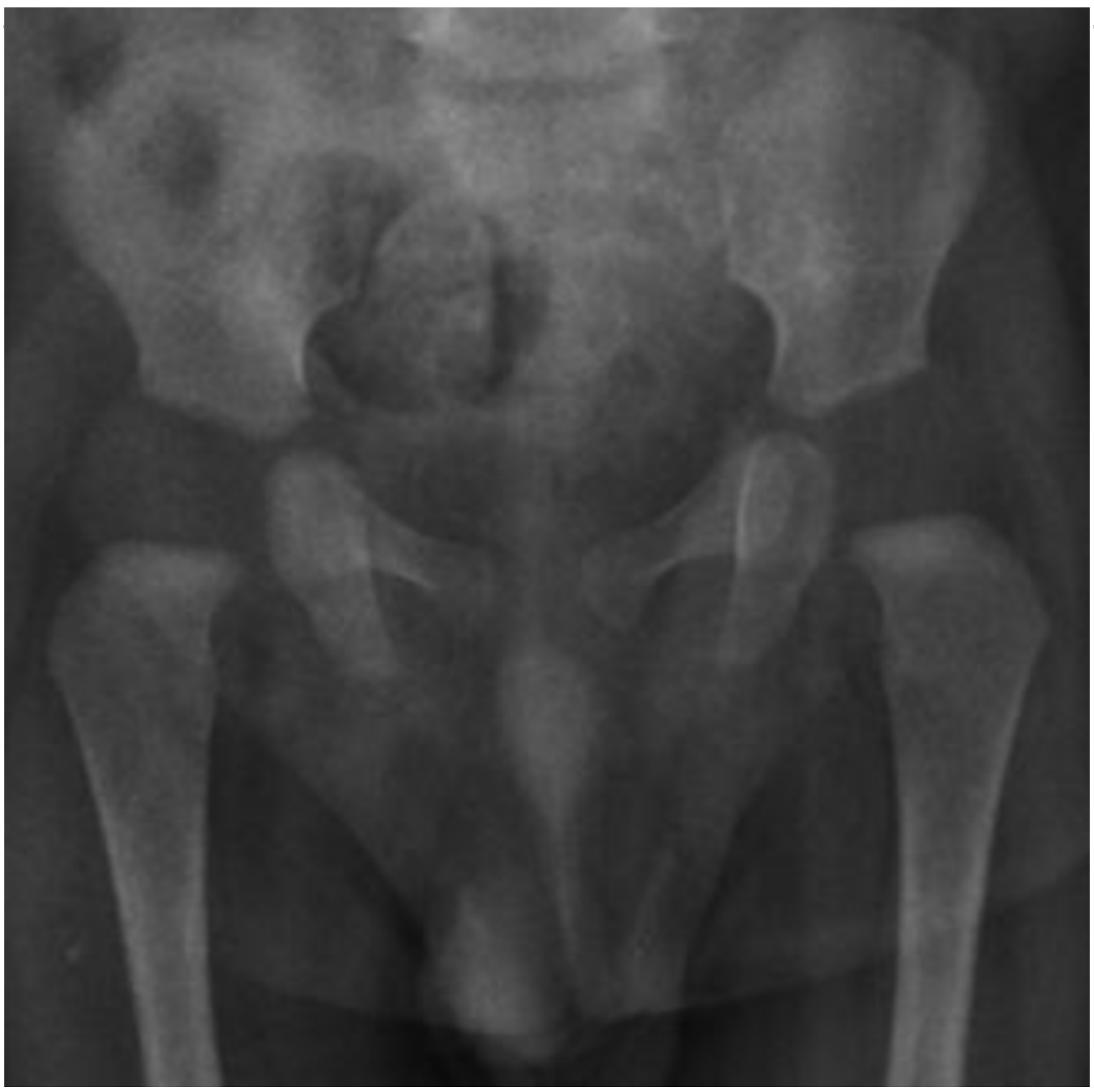

Q10 In relation to the image above, which best describes the pathological appearances in relation to DDH only?

- ◯ Both hips - normal (1)
- ◯ Left hip appearances of DDH - Right hip normal (2)
- ◯ Left hip normal - Right hip appearances of DDH (3)
- ◯ Both hips have appearances of DDH (4)

Q11 Please indicate your confidence level for your decision regarding the presence of DDH. (1=no confidence, 10=full confidence)

	1 2 3 4 5 6 6 7 8 9 10
Confidence rating (1)	

Q12 How would you rate the technical quality (excluding exposure factors) for the above image (for suspected DDH only)?

- ◯ Perfect (1)
- ◯ Good (2)
- ◯ Moderate (3)
- ◯ Inadequate (4)

Page Break

End of Block: Image 1 (N5)

Start of Block: Image 2 (N13)

Q13 (**Image 2**) You are now asked to consider this image of a child with suspected Development Dysplasia of the Hip (DDH). You will be asked to confirm whether this image is **normal** or **abnormal** (**for DDH only**) and indicate a **confidence rating** for your decision (0-not confident, 10-totally confident). You will also be invited to give **your opinion about the image quality**, this should consider ALL elements of image quality BUT NOT those which are exposure factor related (i.e. contrast, density, sharpness, noise).

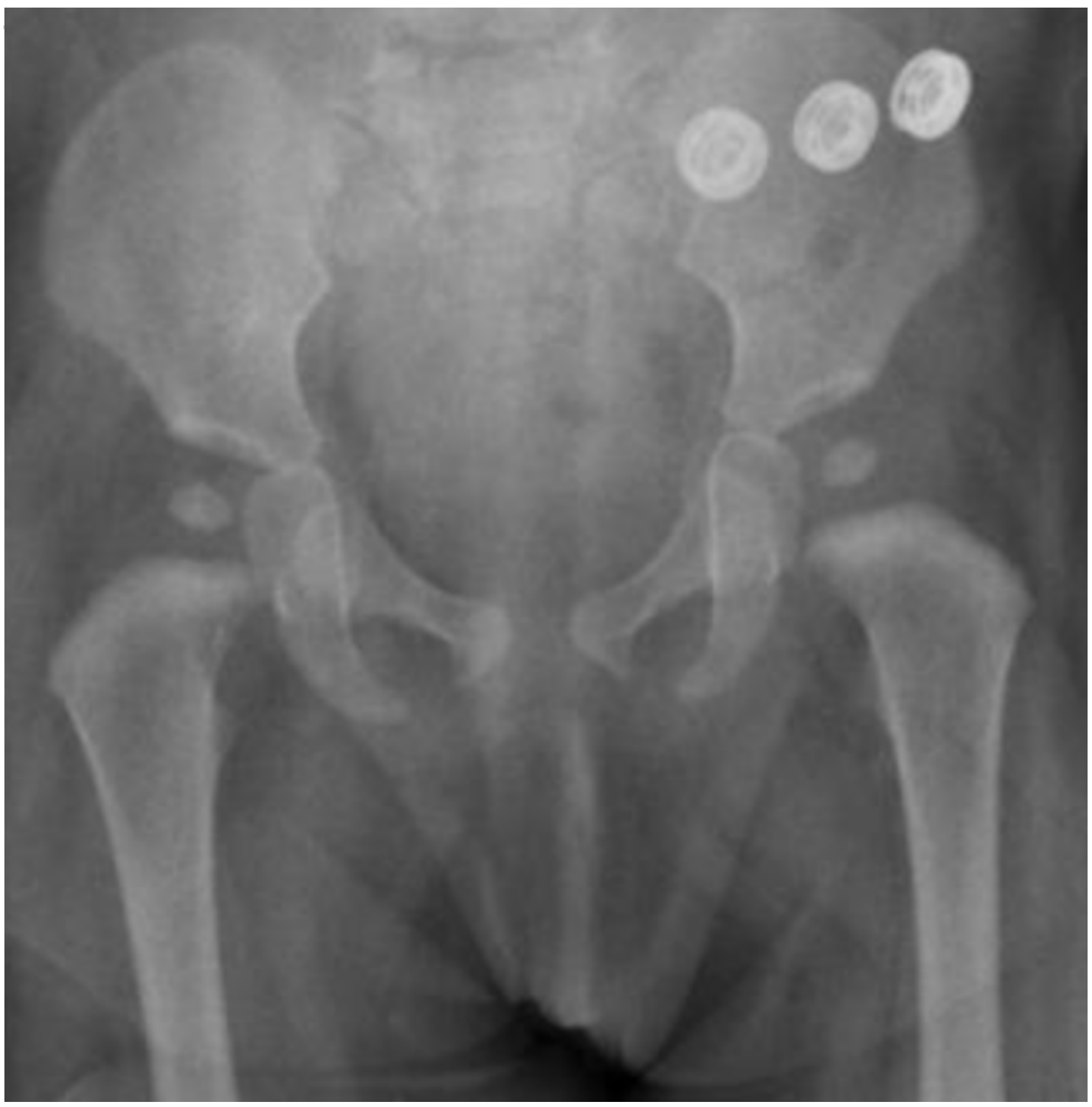

Q14 In relation to the image above, which best describes the pathological appearances in relation to DDH only?

- ◯ Both hips - normal (1)
- ◯ Left hip appearances of DDH - Right hip normal (2)
- ◯ Left hip normal - Right hip appearances of DDH (3)
- ◯ Both hips have appearances of DDH (4)

Q15 Please indicate your confidence level for your decision regarding the presence of DDH. (1=no confidence, 10=full confidence)

	1	2	3	4	5	6	6	7	8	9	10
Confidence rating (1)											

Q16 How would you rate the technical quality (excluding exposure factors) for the above image (for suspected DDH only)?

- ◯ Perfect (1)
- ◯ Good (2)
- ◯ Moderate (3)
- ◯ Inadequate (4)

Page Break

End of Block: Image 2 (N13)

Start of Block: Image 3 (D3)

Q16 (**Image 3**) You are now asked to consider this image of a child with suspected Development Dysplasia of the Hip (DDH). You will asked to confirm whether this image is **normal** or **abnormal** (**for DDH only**) and indicate a **confidence rating** for your decision (0-not confident, 10-totally confident). You will also be invited to give **your opinion about the image quality**, this should consider ALL elements of image quality BUT NOT those which are exposure factor related (i.e. contrast, density, sharpness, noise).

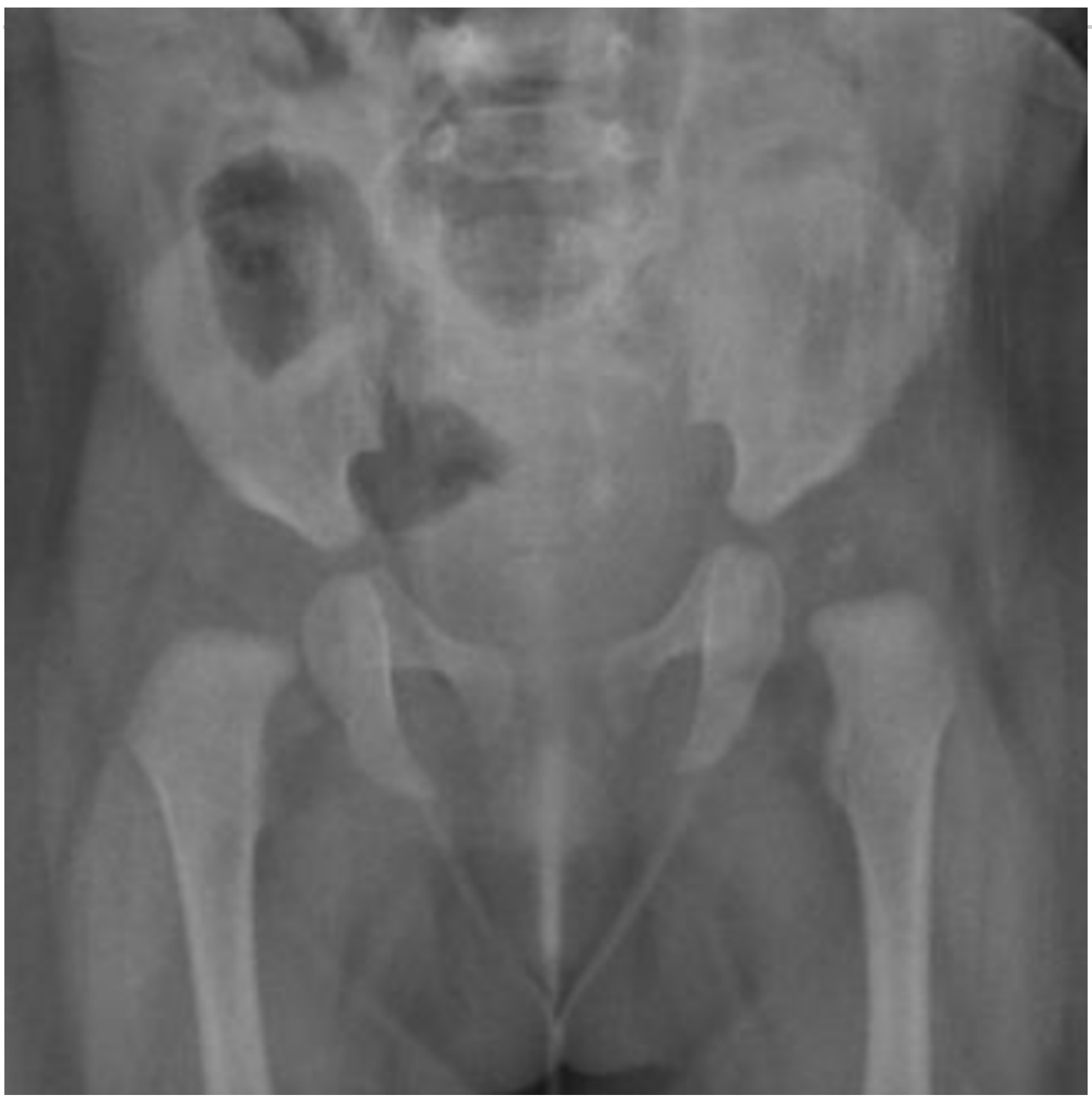

Q17 In relation to the image above, which best describes the pathological appearances in relation to DDH only?

○ Both hips - normal (1)

○ Left hip appearances of DDH - Right hip normal (2)

○ Left hip normal - Right hip appearances of DDH (3)

○ Both hips have appearances of DDH (4)

Q18 Please indicate your confidence level for your decision regarding the presence of DDH. (1=no confidence, 10=full confidence)

	1	2	3	4	5	6	6	7	8	9	10
Confidence rating (1)											

Q19 How would you rate the technical quality (excluding exposure factors) for the above image (for suspected DDH only)?

○ Perfect (1)

○ Good (2)

○ Moderate (3)

○ Inadequate (4)

Page Break

End of Block: Image 3 (D3)

Start of Block: Image 4 (D19)

Q20 (**Image 4**) You are now asked to consider this image of a child with suspected Development Dysplasia of the Hip (DDH). You will asked to confirm whether this image is **normal** or **abnormal** (**for DDH only**) and indicate a **confidence rating** for your decision (0-not confident, 10-totally confident). You will also be invited to give **your opinion about the image quality**, this should consider ALL elements of image quality BUT NOT those which are exposure factor related (i.e. contrast, density, sharpness, noise).

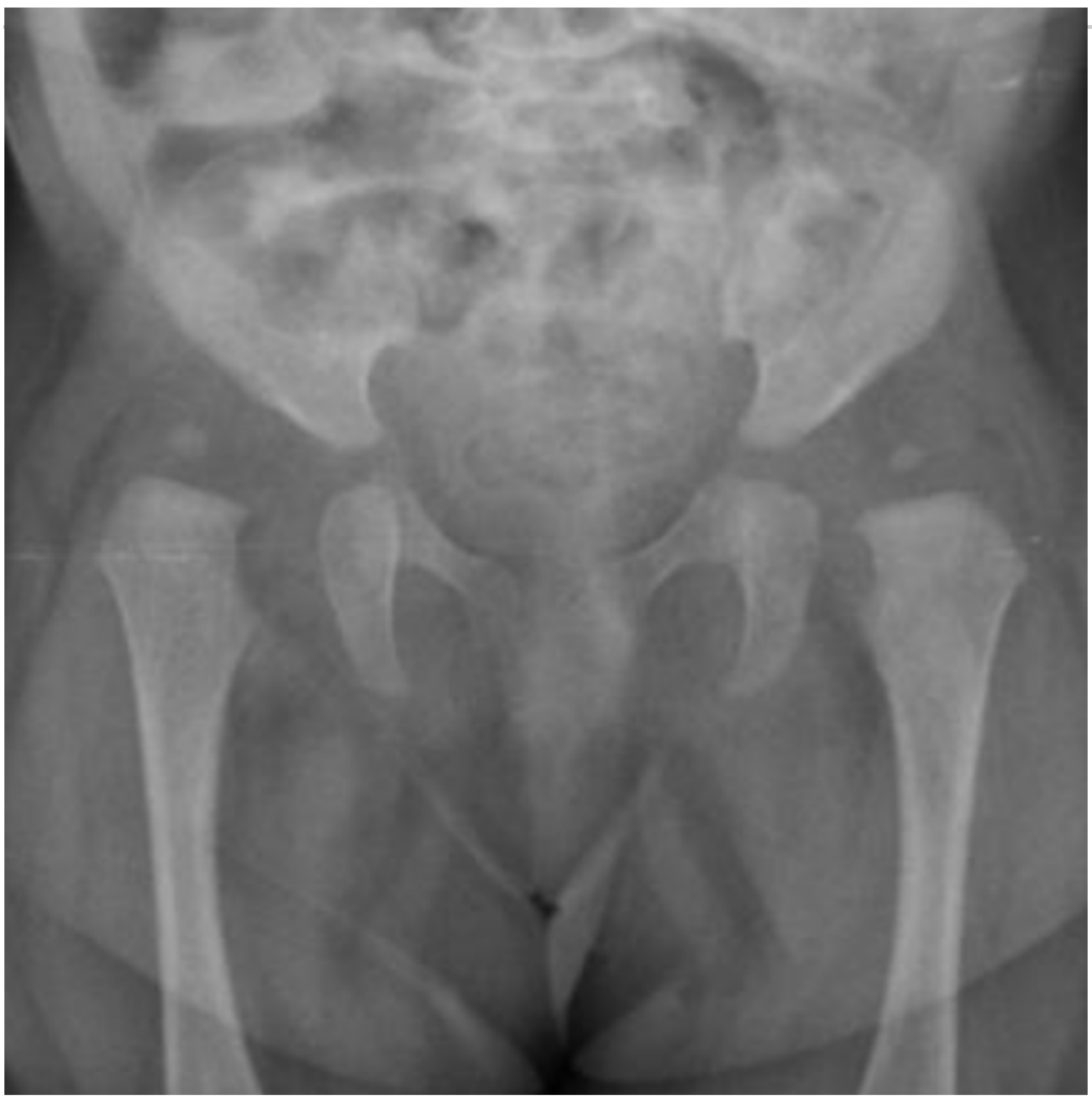

Q21 In relation to the image above, which best describes the pathological appearances in relation to DDH only?

- ◯ Both hips - normal (1)
- ◯ Left hip appearances of DDH - Right hip normal (2)
- ◯ Left hip normal - Right hip appearances of DDH (3)
- ◯ Both hips have appearances of DDH (4)

Q22 Please indicate your confidence level for your decision regarding the presence of DDH. (1=no confidence, 10=full confidence)

	1 2 3 4 5 6 6 7 8 9 10
Confidence rating (1)	

Q23 How would you rate the technical quality (excluding exposure factors) for the above image (for suspected DDH only)?

- ◯ Perfect (1)
- ◯ Good (2)
- ◯ Moderate (3)
- ◯ Inadequate (4)

Page Break

End of Block: Image 4 (D19)

Start of Block: Image 5 (D35)

Q24 (**Image 5**) You are now asked to consider this image of a child with suspected Development Dysplasia of the Hip (DDH). You will asked to confirm whether this image is **normal** or **abnormal** (**for DDH only**) and indicate a **confidence rating** for your decision (0-not confident, 10-totally confident). You will also be invited to give **your opinion about the image quality**, this should consider ALL elements of image quality BUT NOT those which are exposure factor related (i.e. contrast, density, sharpness, noise).

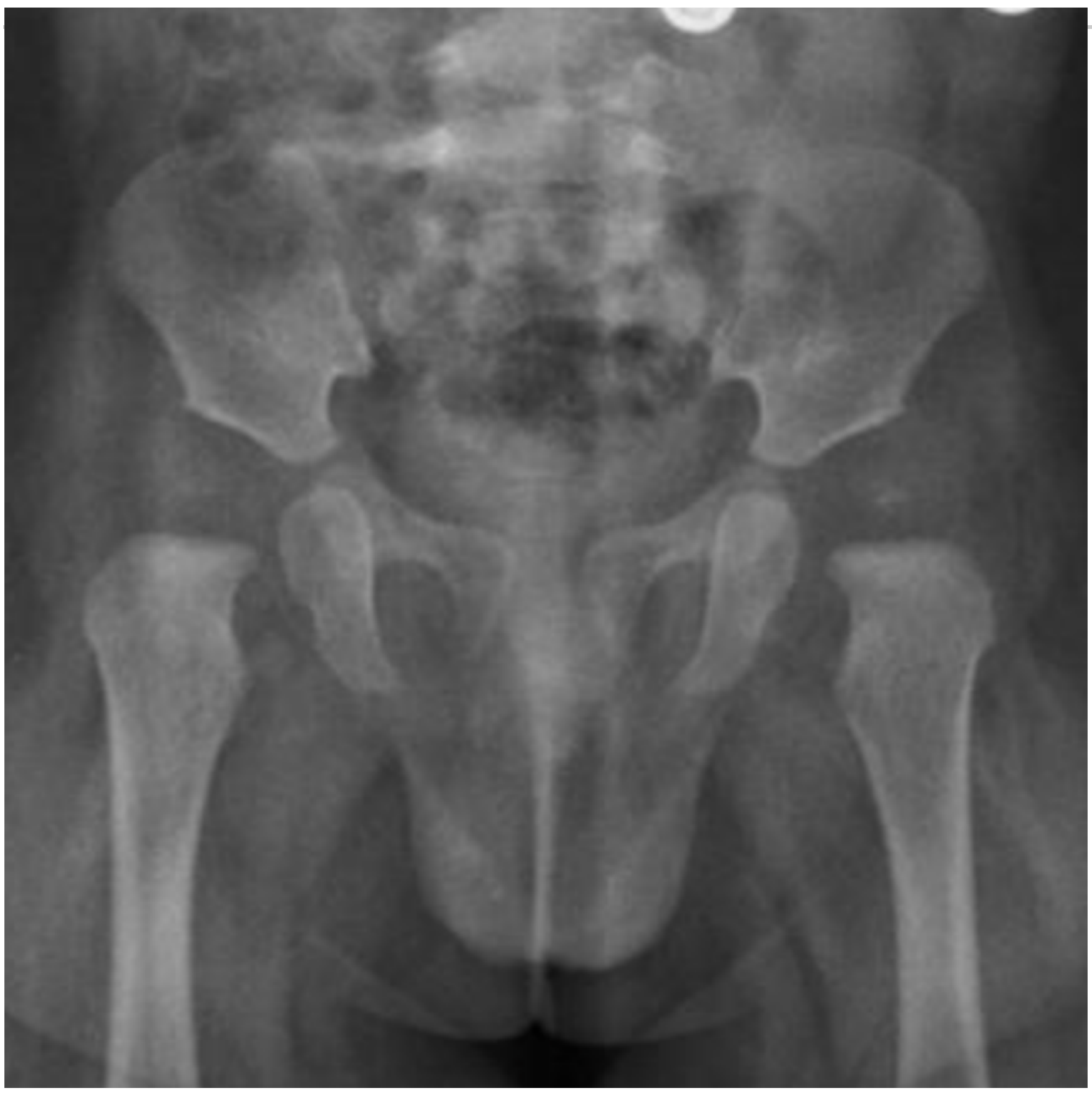

Q25 In relation to the image above, which best describes the pathological appearances in relation to DDH only?

◯ Both hips - normal (1)

◯ Left hip appearances of DDH - Right hip normal (2)

◯ Left hip normal - Right hip appearances of DDH (3)

◯ Both hips have appearances of DDH (4)

Q26 Please indicate your confidence level for your decision regarding the presence of DDH. (1=no confidence, 10=full confidence)

	1 2 3 4 5 6 6 7 8 9 10
Confidence rating (1)	

Q27 How would you rate the technical quality (excluding exposure factors) for the above image (for suspected DDH only)?

◯ Perfect (1)

◯ Good (2)

◯ Moderate (3)

◯ Inadequate (4)

Page Break

End of Block: Image 5 (D35)

Start of Block: Image 6 (N50)

Q28 (**Image 6**) You are now asked to consider this image of a child with suspected Development Dysplasia of the Hip (DDH). You will asked to confirm whether this image is **normal** or **abnormal** (**for DDH only**) and indicate a **confidence rating** for your decision (0-not confident, 10-totally confident). You will also be invited to give **your opinion about the image quality**, this should consider ALL elements of image quality BUT NOT those which are exposure factor related (i.e. contrast, density, sharpness, noise).

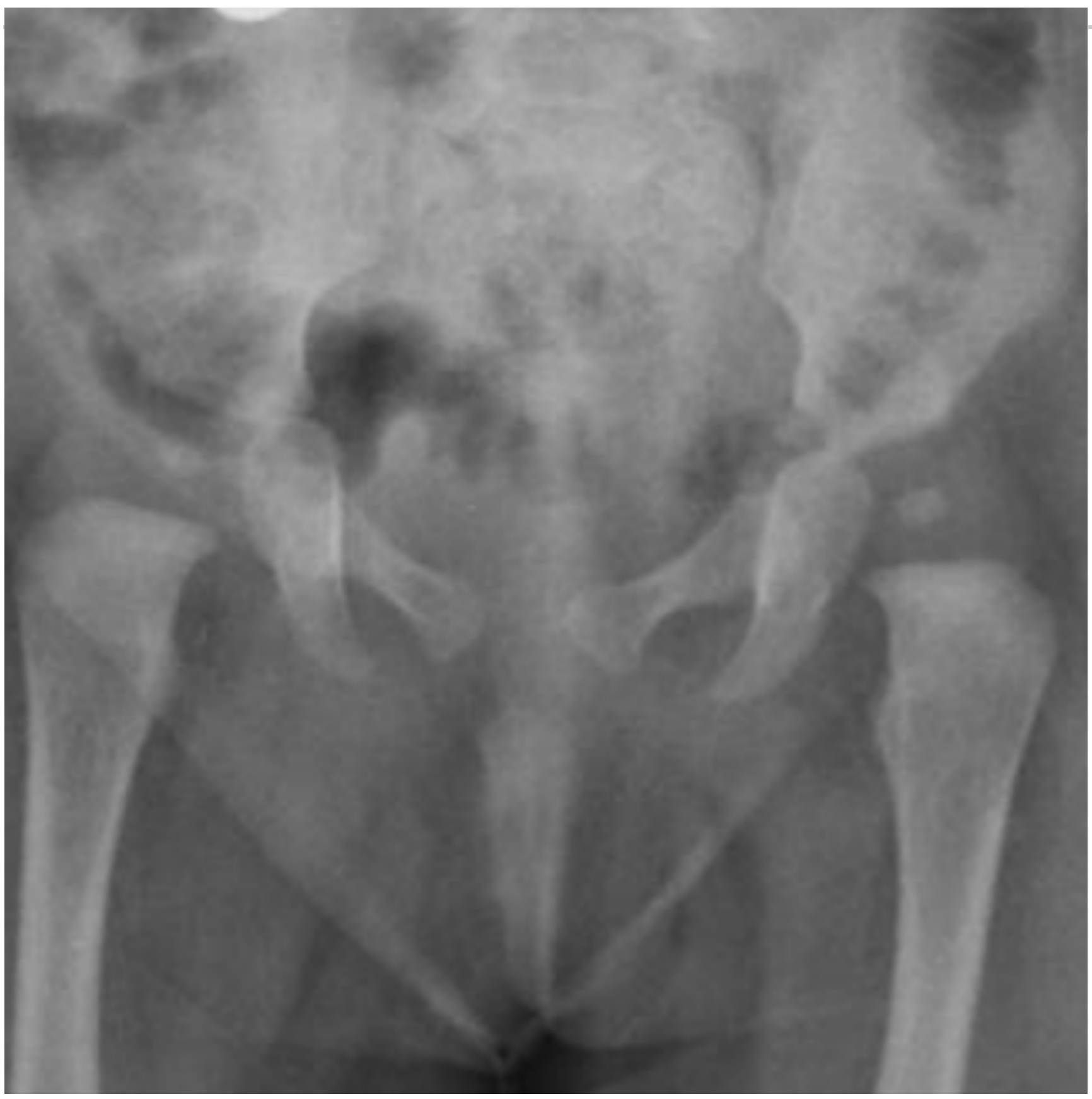

Q29 In relation to the image above, which best describes the pathological appearances in relation to DDH only?

- ◯ Both hips - normal (1)
- ◯ Left hip appearances of DDH - Right hip normal (2)
- ◯ Left hip normal - Right hip appearances of DDH (3)
- ◯ Both hips have appearances of DDH (4)

Q30 Please indicate your confidence level for your decision regarding the presence of DDH. (1=no confidence, 10=full confidence)

	1 2 3 4 5 6 6 7 8 9 10
Confidence rating (1)	

Q31 How would you rate the technical quality (excluding exposure factors) for the above image (for suspected DDH only)?

- ◯ Perfect (1)
- ◯ Good (2)
- ◯ Moderate (3)
- ◯ Inadequate (4)

Page Break

End of Block: Image 6 (N50)

Start of Block: Image 7 (D115)

Q32 (**Image 7**) You are now asked to consider this image of a child with suspected Development Dysplasia of the Hip (DDH). You will asked to confirm whether this image is **normal** or **abnormal** (**for DDH only**) and indicate a **confidence rating** for your decision (0-not confident, 10-totally confident). You will also be invited to give **your opinion about the image quality**, this should consider ALL elements of image quality BUT NOT those which are exposure factor related (i.e. contrast, density, sharpness, noise).

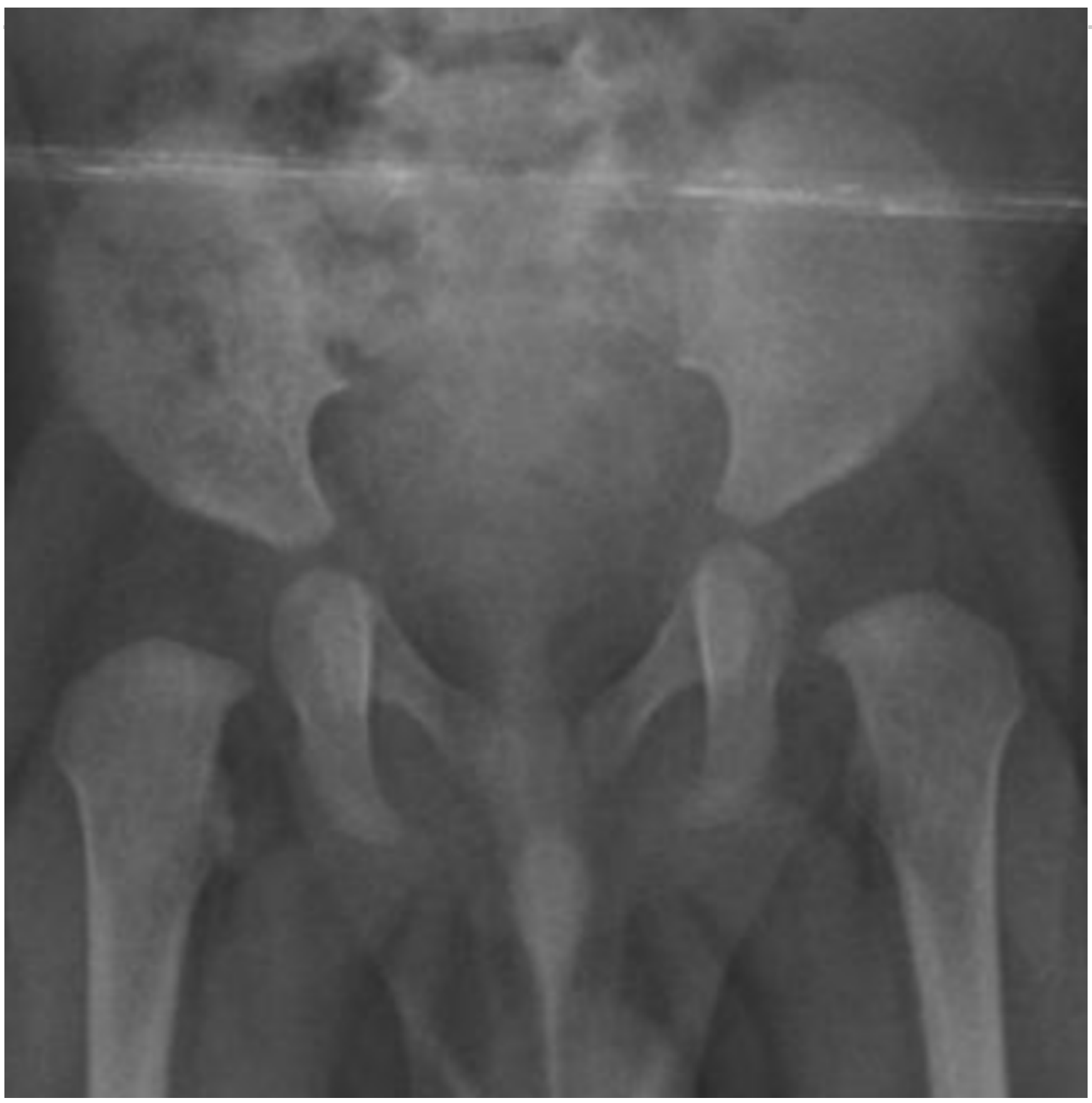

Q33 In relation to the image above, which best describes the pathological appearances in relation to DDH only?

- ○ Both hips - normal (1)
- ○ Left hip appearances of DDH - Right hip normal (2)
- ○ Left hip normal - Right hip appearances of DDH (3)
- ○ Both hips have appearances of DDH (4)

Q34 Please indicate your confidence level for your decision regarding the presence of DDH. (1=no confidence, 10=full confidence)

	1	2	3	4	5	6	6	7	8	9	10
Confidence rating (1)											

Q35 How would you rate the technical quality (excluding exposure factors) for the above image (for suspected DDH only)?

- ○ Perfect (1)
- ○ Good (2)
- ○ Moderate (3)
- ○ Inadequate (4)

Page Break

End of Block: Image 7 (D115)

Start of Block: Image 8 (N82)

Q36 (**Image 8**) You are now asked to consider this image of a child with suspected Development Dysplasia of the Hip (DDH). You will asked to confirm whether this image is **normal** or **abnormal** (**for DDH only**) and indicate a **confidence rating** for your decision (0-not confident, 10-totally confident). You will also be invited to give **your opinion about the image quality**, this should consider ALL elements of image quality BUT NOT those which are exposure factor related (i.e. contrast, density, sharpness, noise).

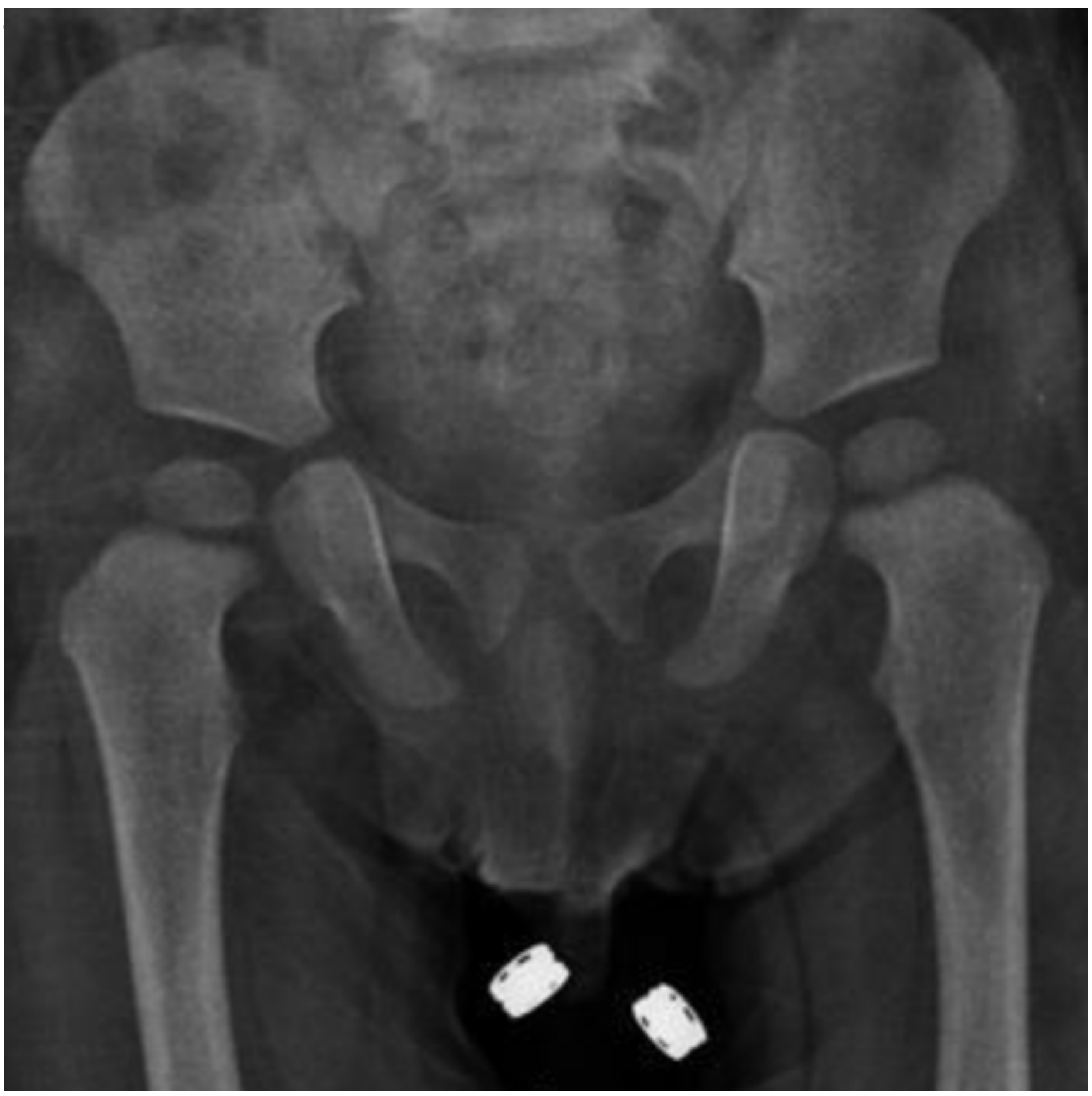

Q37 In relation to the image above, which best describes the pathological appearances in relation to DDH only?

- ◯ Both hips - normal (1)
- ◯ Left hip appearances of DDH - Right hip normal (2)
- ◯ Left hip normal - Right hip appearances of DDH (3)
- ◯ Both hips have appearances of DDH (4)

Q38 Please indicate your confidence level for your decision regarding the presence of DDH. (1=no confidence, 10=full confidence)

	1	2	3	4	5	6	6	7	8	9	10
Confidence rating (1)											

Q39 How would you rate the technical quality (excluding exposure factors) for the above image (for suspected DDH only)?

- ◯ Perfect (1)
- ◯ Good (2)
- ◯ Moderate (3)
- ◯ Inadequate (4)

Page Break

End of Block: Image 8 (N82)

Start of Block: Image 9 (N90)

Q40 (**Image 9**) You are now asked to consider this image of a child with suspected Development Dysplasia of the Hip (DDH). You will asked to confirm whether this image is **normal** or **abnormal** (**for DDH only**) and indicate a **confidence rating** for your decision (0-not confident, 10-totally confident). You will also be invited to give **your opinion about the image quality**, this should consider ALL elements of image quality BUT NOT those which are exposure factor related (i.e. contrast, density, sharpness, noise).

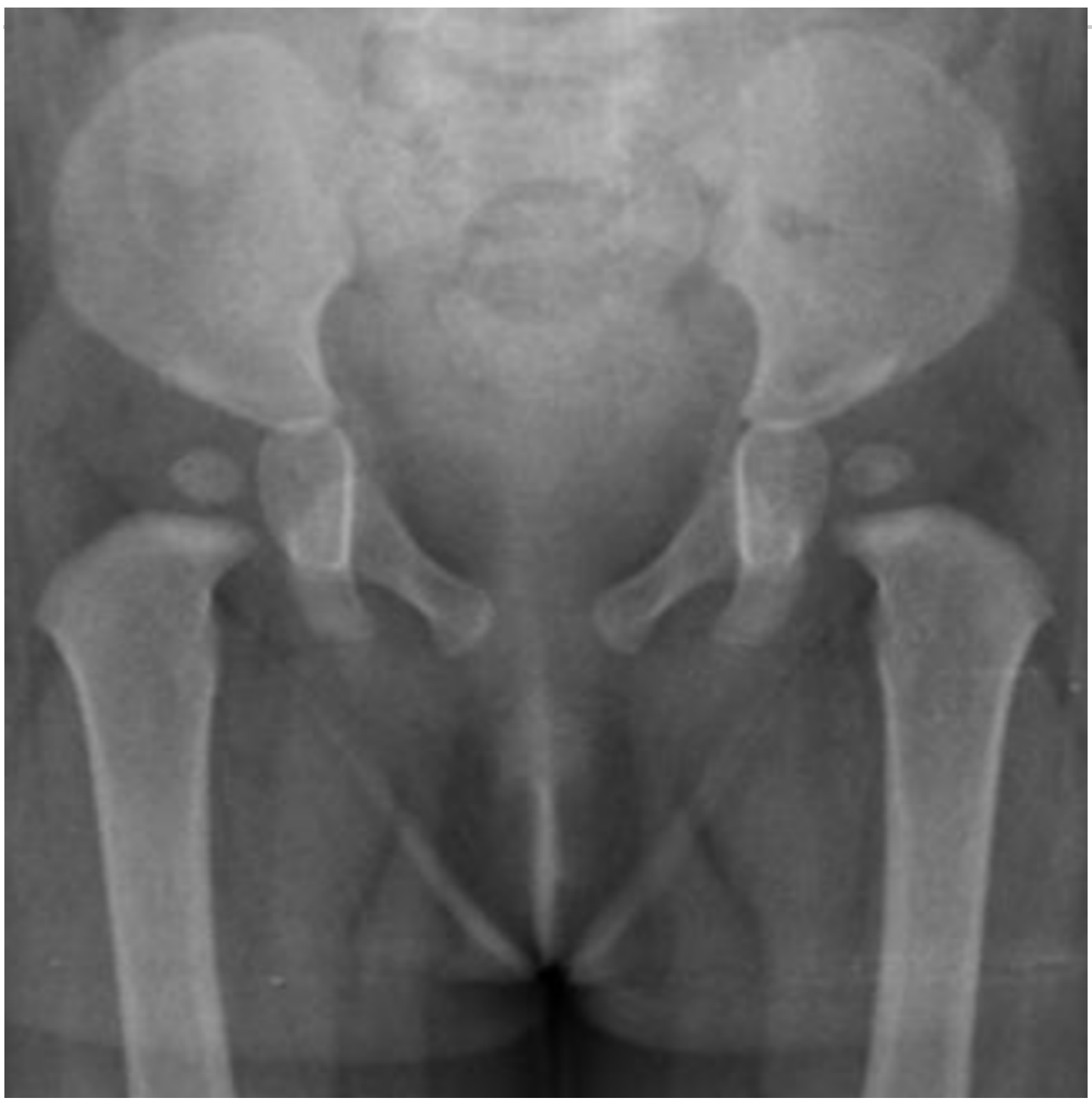

Q41 In relation to the image above, which best describes the pathological appearances in relation to DDH only?

- ○ Both hips - normal (1)
- ○ Left hip appearances of DDH - Right hip normal (2)
- ○ Left hip normal - Right hip appearances of DDH (3)
- ○ Both hips have appearances of DDH (4)

Q42 Please indicate your confidence level for your decision regarding the presence of DDH. (1=no confidence, 10=full confidence)

	1	2	3	4	5	6	6	7	8	9	10
Confidence rating (1)											

Q43 How would you rate the technical quality (excluding exposure factors) for the above image (for suspected DDH only)?

- ○ Perfect (1)
- ○ Good (2)
- ○ Moderate (3)
- ○ Inadequate (4)

Page Break

End of Block: Image 9 (N90)

Start of Block: Image 10 (D240)

Q44 (**Image 10**) You are now asked to consider this image of a child with suspected Development Dysplasia of the Hip (DDH). You will asked to confirm whether this image is **normal** or **abnormal** (**for DDH only**) and indicate a **confidence rating** for your decision (0-not confident, 10-totally confident). You will also be invited to give **your opinion about the image quality**, this should consider ALL elements of image quality BUT NOT those which are exposure factor related (i.e. contrast, density, sharpness, noise).

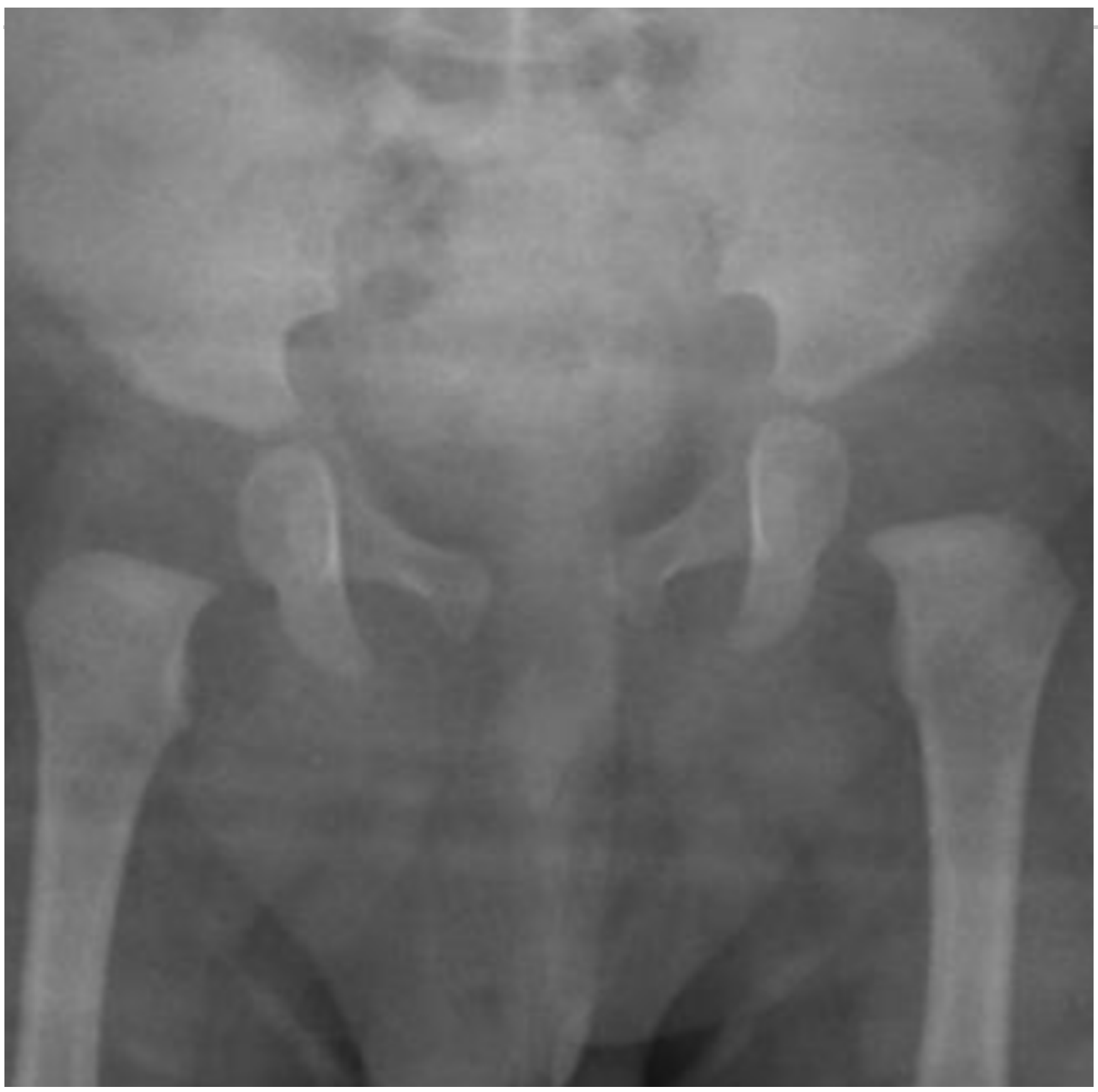

Q45 In relation to the image above, which best describes the pathological appearances in relation to DDH only?

- ◯ Both hips - normal (1)
- ◯ Left hip appearances of DDH - Right hip normal (2)
- ◯ Left hip normal - Right hip appearances of DDH (3)
- ◯ Both hips have appearances of DDH (4)

Q46 Please indicate your confidence level for your decision regarding the presence of DDH. (1=no confidence, 10=full confidence)

	1	2	3	4	5	6	6	7	8	9	10
Confidence rating (1)											

Q47 How would you rate the technical quality (excluding exposure factors) for the above image (for suspected DDH only)?

- ◯ Perfect (1)
- ◯ Good (2)
- ◯ Moderate (3)
- ◯ Inadequate (4)

Page Break

End of Block: Image 10 (D240)

Start of Block: Image 11 (D191)

Q48 (**Image 11**) You are now asked to consider this image of a child with suspected Development Dysplasia of the Hip (DDH). You will asked to confirm whether this image is **normal** or **abnormal** (**for DDH only**) and indicate a **confidence rating** for your decision (0-not confident, 10-totally confident). You will also be invited to give **your opinion about the image quality**, this should consider ALL elements of image quality BUT NOT those which are exposure factor related (i.e. contrast, density, sharpness, noise).

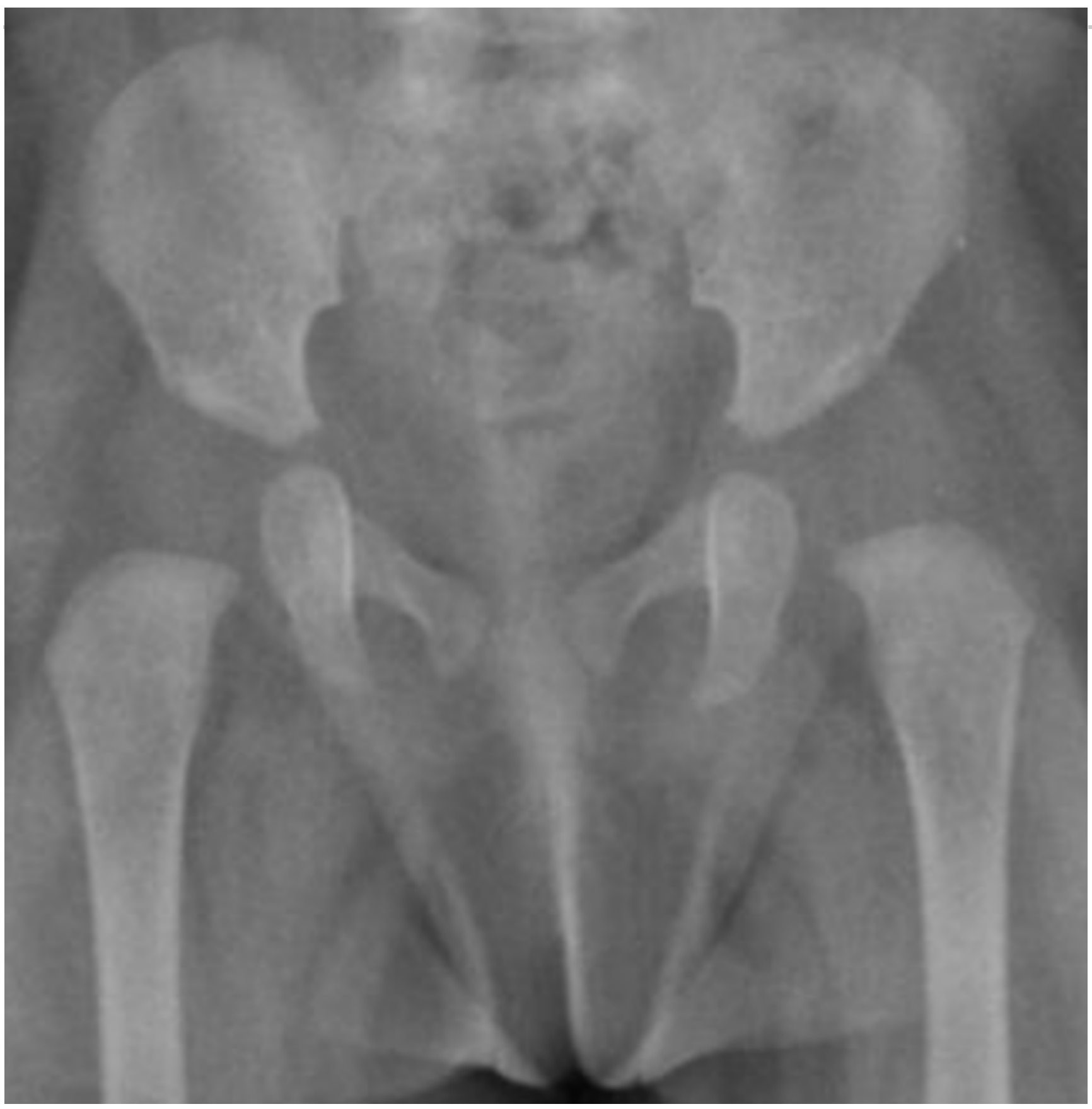

Q49 In relation to the image above, which best describes the pathological appearances in relation to DDH only?

- ◯ Both hips - normal (1)
- ◯ Left hip appearances of DDH - Right hip normal (2)
- ◯ Left hip normal - Right hip appearances of DDH (3)
- ◯ Both hips have appearances of DDH (4)

Q50 Please indicate your confidence level for your decision regarding the presence of DDH. (1=no confidence, 10=full confidence)

	1 2 3 4 5 6 6 7 8 9 10
Confidence rating (1)	

Q51 How would you rate the technical quality (excluding exposure factors) for the above image (for suspected DDH only)?

- ◯ Perfect (1)
- ◯ Good (2)
- ◯ Moderate (3)
- ◯ Inadequate (4)

Page Break

End of Block: Image 11 (D191)

Start of Block: Image 12 (D97)

Q52 (**Image 12**) You are now asked to consider this image of a child with suspected Development Dysplasia of the Hip (DDH). You will asked to confirm whether this image is **normal** or **abnormal** (**for DDH only**) and indicate a **confidence rating** for your decision (0-not confident, 10-totally confident). You will also be invited to give **your opinion about the image quality**, this should consider ALL elements of image quality BUT NOT those which are exposure factor related (i.e. contrast, density, sharpness, noise).

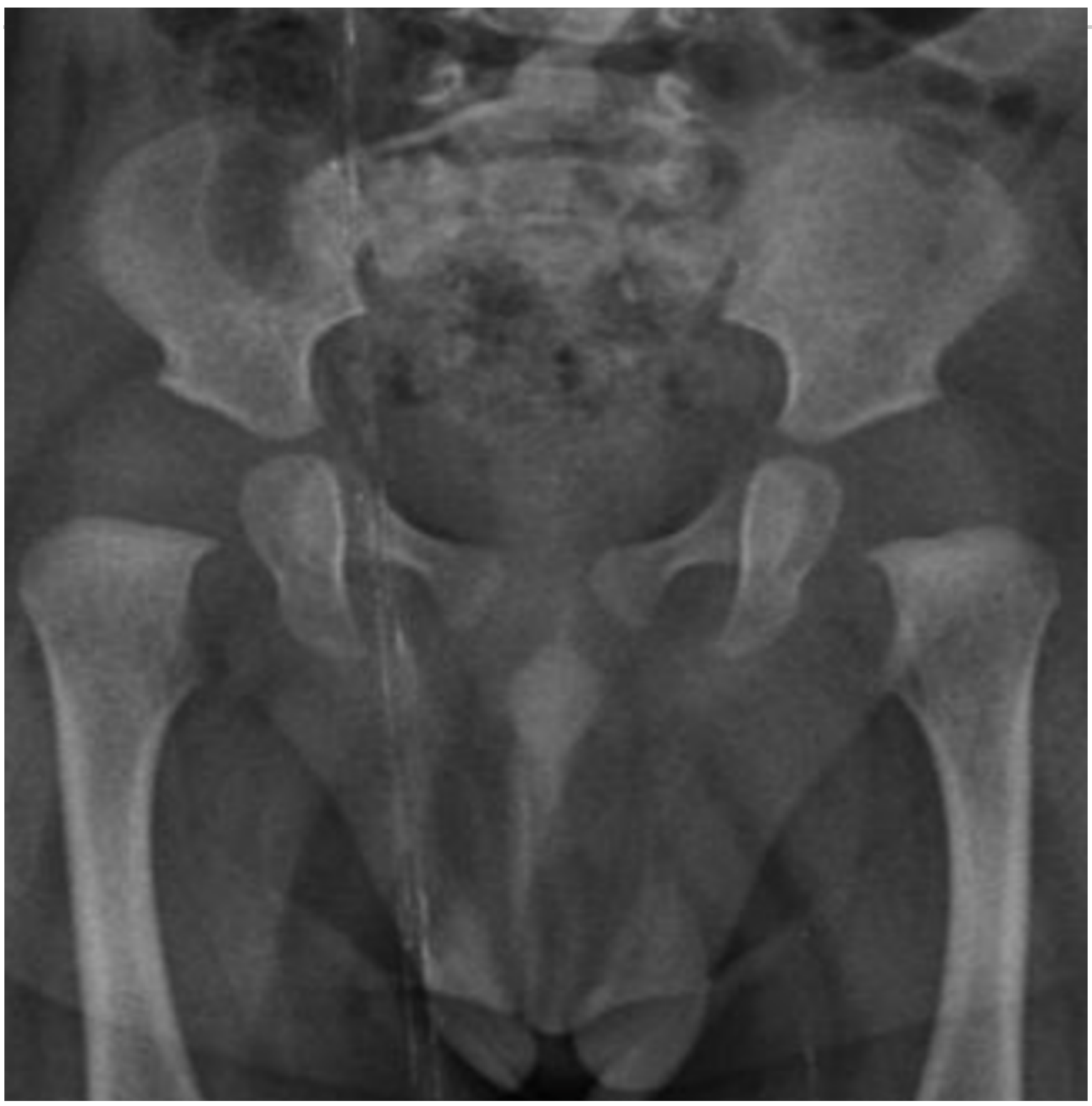

Q53 In relation to the image above, which best describes the pathological appearances in relation to DDH only?

- ◯ Both hips - normal (1)
- ◯ Left hip appearances of DDH - Right hip normal (2)
- ◯ Left hip normal - Right hip appearances of DDH (3)
- ◯ Both hips have appearances of DDH (4)

Q54 Please indicate your confidence level for your decision regarding the presence of DDH. (1=no confidence, 10=full confidence)

	1	2	3	4	5	6	6	7	8	9	10
Confidence rating (1)											

Q55 How would you rate the technical quality (excluding exposure factors) for the above image (for suspected DDH only)?

- ◯ Perfect (1)
- ◯ Good (2)
- ◯ Moderate (3)
- ◯ Inadequate (4)

Page Break

End of Block: Image 12 (D97)

Start of Block: Image 13 (N304)

Q56 (**Image 13**) You are now asked to consider this image of a child with suspected Development Dysplasia of the Hip (DDH). You will asked to confirm whether this image is **normal** or **abnormal** (**for DDH only**) and indicate a **confidence rating** for your decision (0-not confident, 10-totally confident). You will also be invited to give **your opinion about the image quality**, this should consider ALL elements of image quality BUT NOT those which are exposure factor related (i.e. contrast, density, sharpness, noise).

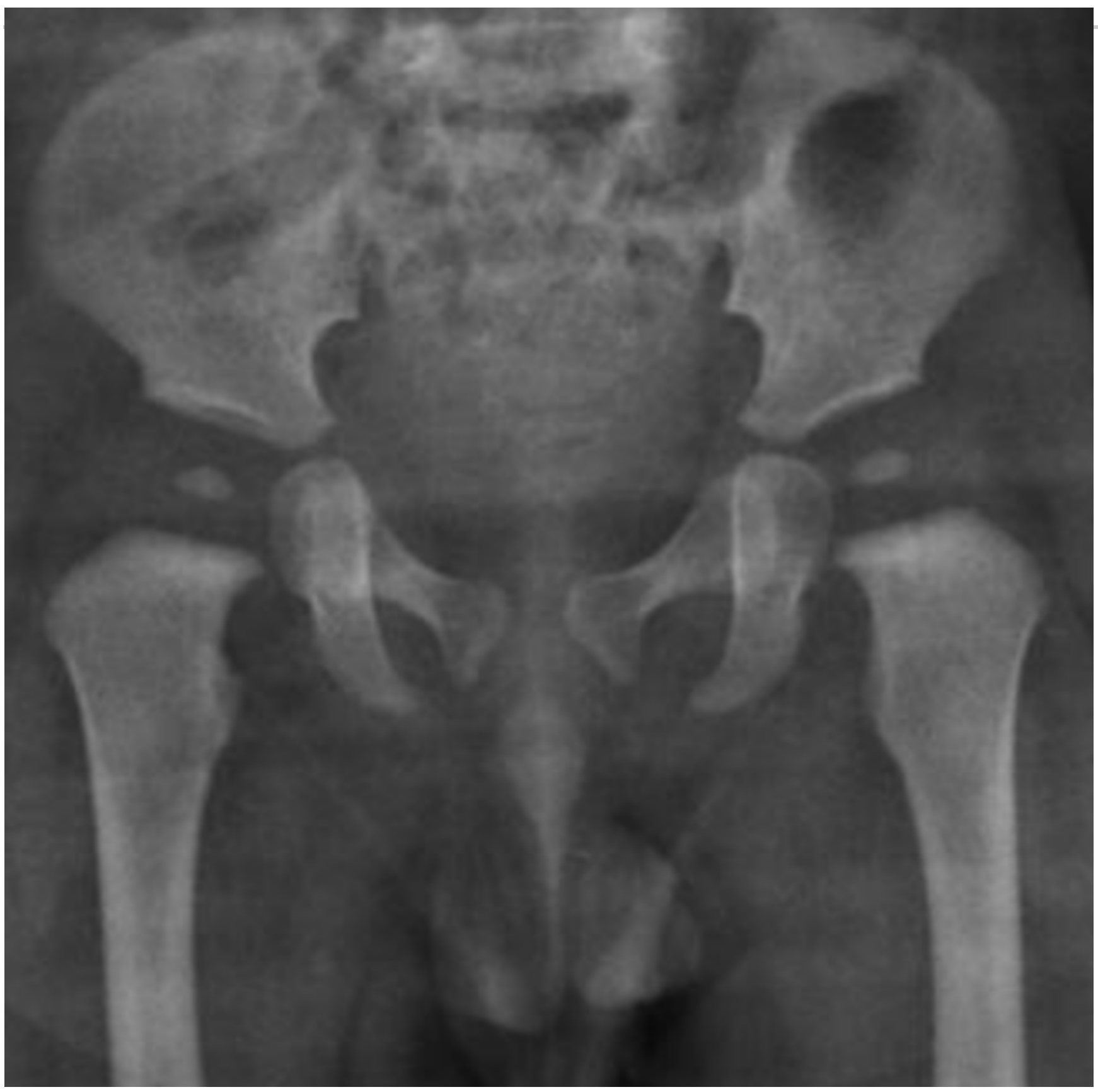

Q57 In relation to the image above, which best describes the pathological appearances in relation to DDH only?

- ◯ Both hips - normal (1)
- ◯ Left hip appearances of DDH - Right hip normal (2)
- ◯ Left hip normal - Right hip appearances of DDH (3)
- ◯ Both hips have appearances of DDH (4)

Q58 Please indicate your confidence level for your decision regarding the presence of DDH. (1=no confidence, 10=full confidence)

	1	2	3	4	5	6	6	7	8	9	10
Confidence rating (1)											

Q59 How would you rate the technical quality (excluding exposure factors) for the above image (for suspected DDH only)?

- ◯ Perfect (1)
- ◯ Good (2)
- ◯ Moderate (3)
- ◯ Inadequate (4)

Page Break

End of Block: Image 13 (N304)

Start of Block: Image 14 (D74)

Q60 (**Image 14**) You are now asked to consider this image of a child with suspected Development Dysplasia of the Hip (DDH). You will asked to confirm whether this image is **normal** or **abnormal** (**for DDH only**) and indicate a **confidence rating** for your decision (0-not confident, 10-totally confident). You will also be invited to give **your opinion about the image quality**, this should consider ALL elements of image quality BUT NOT those which are exposure factor related (i.e. contrast, density, sharpness, noise).

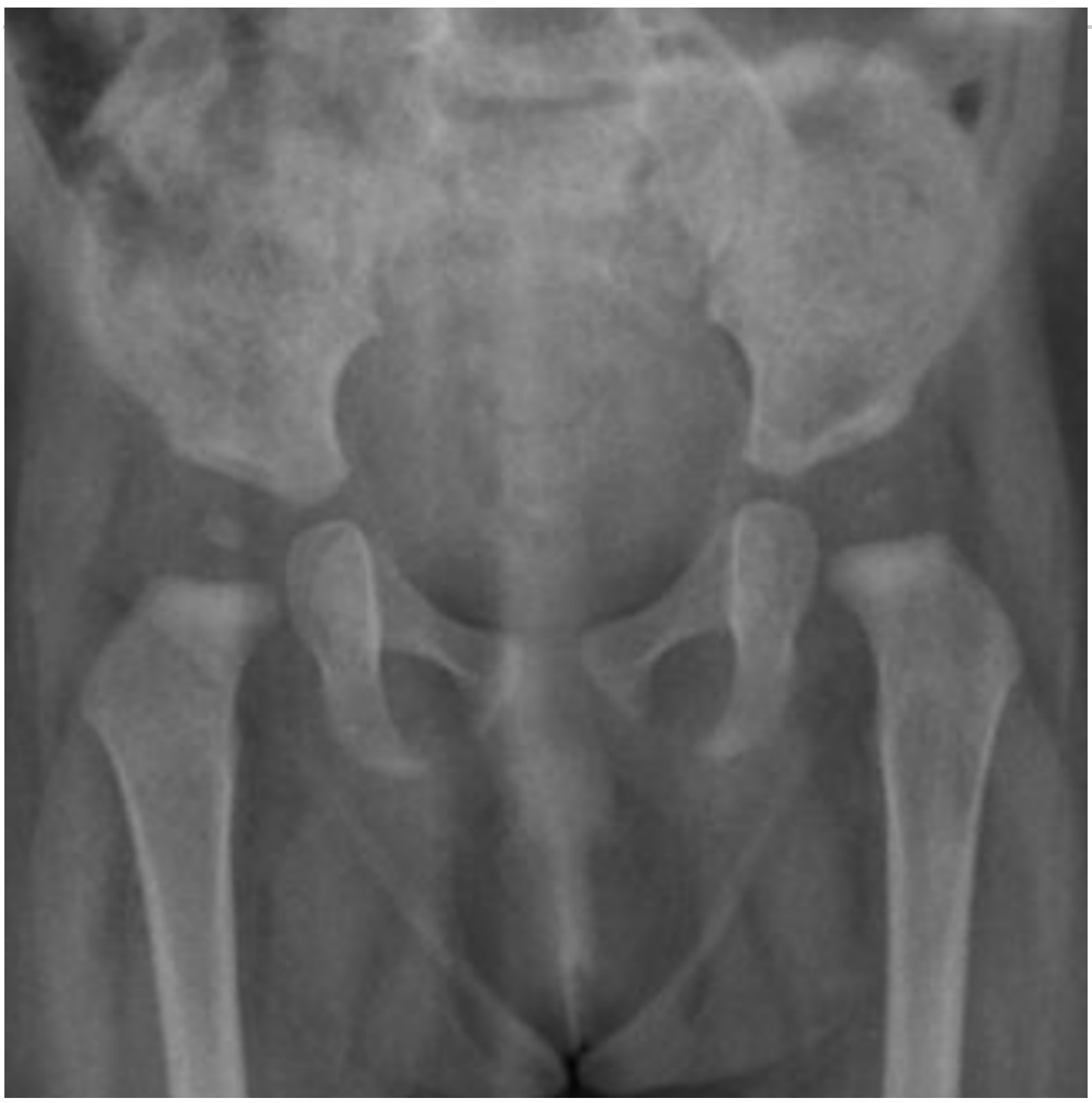

Q61 In relation to the image above, which best describes the pathological appearances in relation to DDH only?

- ◯ Both hips - normal (1)
- ◯ Left hip appearances of DDH - Right hip normal (2)
- ◯ Left hip normal - Right hip appearances of DDH (3)
- ◯ Both hips have appearances of DDH (4)

Q62 Please indicate your confidence level for your decision regarding the presence of DDH. (1=no confidence, 10=full confidence)

	1	2	3	4	5	6	6	7	8	9	10
Confidence rating (1)											

Q63 How would you rate the technical quality (excluding exposure factors) for the above image (for suspected DDH only)?

- ◯ Perfect (1)
- ◯ Good (2)
- ◯ Moderate (3)
- ◯ Inadequate (4)

Page Break

End of Block: Image 14 (D74)

Start of Block: Image 15 (N16)

Q64 **(Image 15**) You are now asked to consider this image of a child with suspected Development Dysplasia of the Hip (DDH). You will asked to confirm whether this image is **normal** or **abnormal** (**for DDH only**) and indicate a **confidence rating** for your decision (0-not confident, 10-totally confident). You will also be invited to give **your opinion about the image quality**, this should consider ALL elements of image quality BUT NOT those which are exposure factor related (i.e. contrast, density, sharpness, noise).

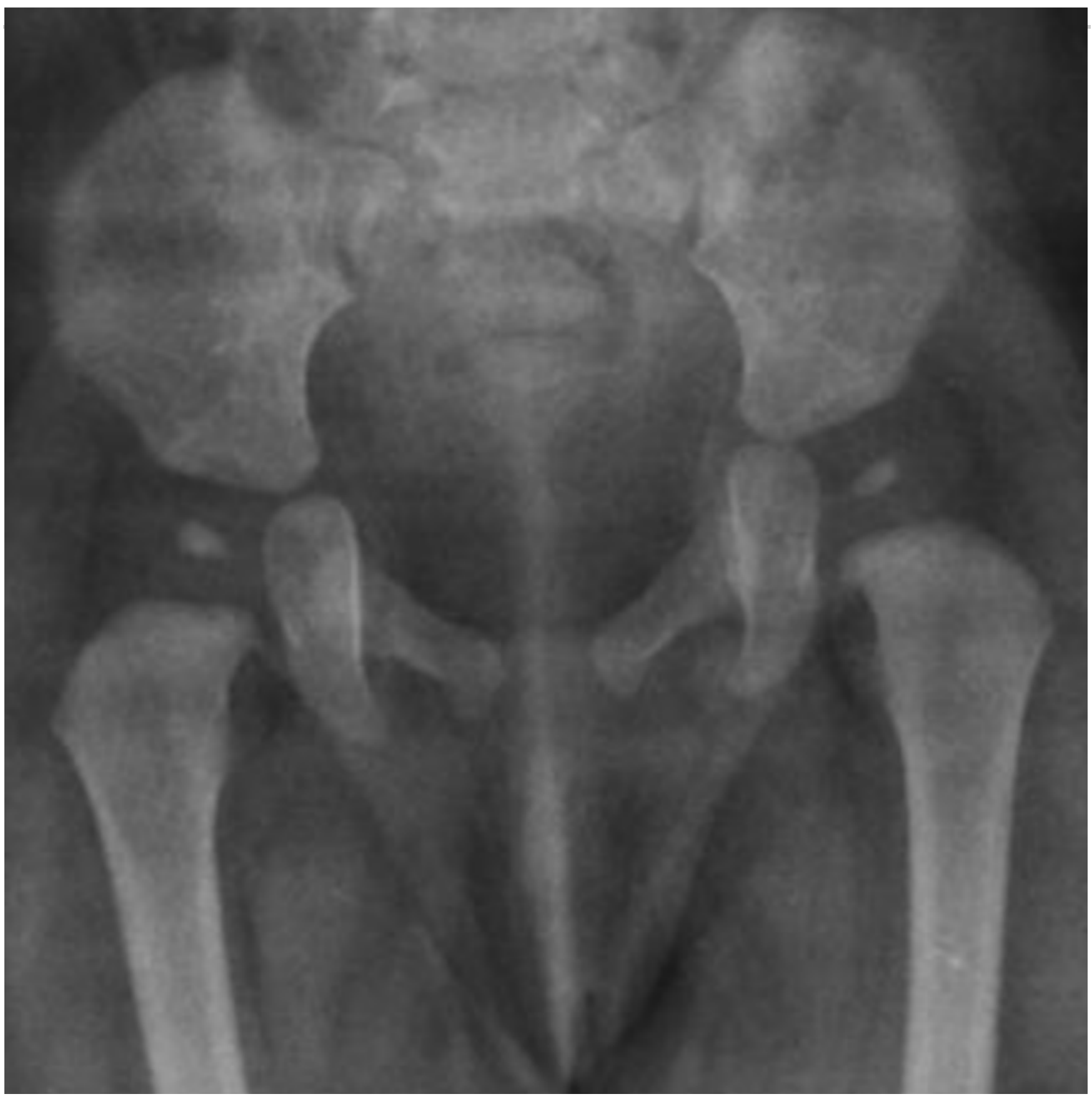

Q65 In relation to the image above, which best describes the pathological appearances in relation to DDH only?

- ◯ Both hips - normal (1)
- ◯ Left hip appearances of DDH - Right hip normal (2)
- ◯ Left hip normal - Right hip appearances of DDH (3)
- ◯ Both hips have appearances of DDH (4)

Q66 Please indicate your confidence level for your decision regarding the presence of DDH. (1=no confidence, 10=full confidence)

	1	2	3	4	5	6	6	7	8	9	10
Confidence rating (1)											

Q67 How would you rate the technical quality (excluding exposure factors) for the above image (for suspected DDH only)?

- ◯ Perfect (1)
- ◯ Good (2)
- ◯ Moderate (3)
- ◯ Inadequate (4)

Page Break

End of Block: Image 15 (N16)

Start of Block: Image 16 (N20)

Q68 (**Image 16**) You are now asked to consider this image of a child with suspected Development Dysplasia of the Hip (DDH). You will asked to confirm whether this image is **normal** or **abnormal** (**for DDH only**) and indicate a **confidence rating** for your decision (0-not confident, 10-totally confident). You will also be invited to give **your opinion about the image quality**, this should consider ALL elements of image quality BUT NOT those which are exposure factor related (i.e. contrast, density, sharpness, noise).

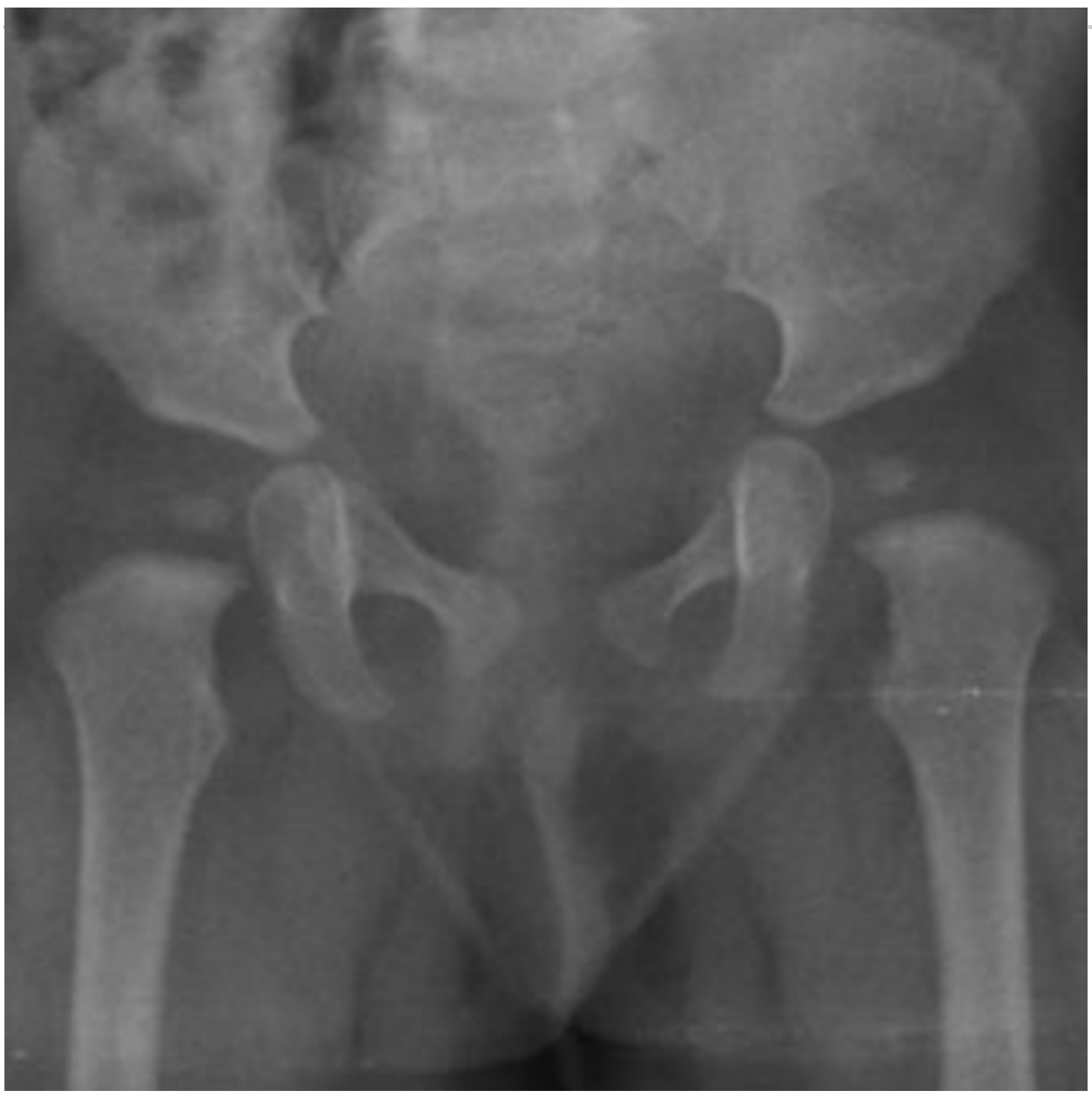

Q69 In relation to the image above, which best describes the pathological appearances in relation to DDH only?

- Both hips - normal (1)
- Left hip appearances of DDH - Right hip normal (2)
- Left hip normal - Right hip appearances of DDH (3)
- Both hips have appearances of DDH (4)

Q70 Please indicate your confidence level for your decision regarding the presence of DDH. (1=no confidence, 10=full confidence)

	1 2 3 4 5 6 6 7 8 9 10
Confidence rating (1)	

Q71 How would you rate the technical quality (excluding exposure factors) for the above image (for suspected DDH only)?

- Perfect (1)
- Good (2)
- Moderate (3)
- Inadequate (4)

Page Break

End of Block: Image 16 (N20)

Start of Block: Image 17 (N50)

Q72 (**Image 17**) You are now asked to consider this image of a child with suspected Development Dysplasia of the Hip (DDH). You will asked to confirm whether this image is **normal** or **abnormal** (**for DDH only**) and indicate a **confidence rating** for your decision (0-not confident, 10-totally confident). You will also be invited to give **your opinion about the image quality**, this should consider ALL elements of image quality BUT NOT those which are exposure factor related (i.e. contrast, density, sharpness, noise).

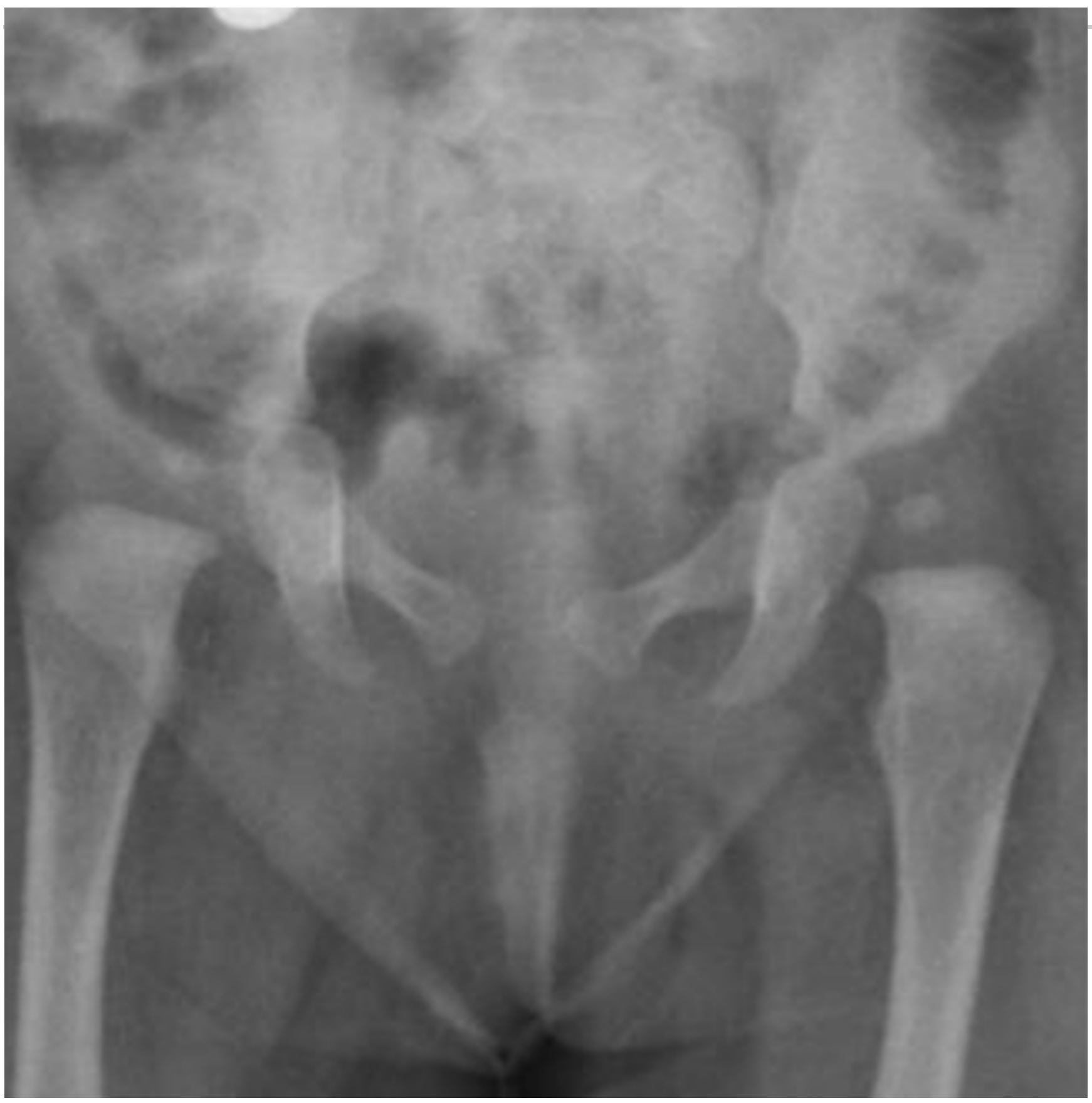

Q73 In relation to the image above, which best describes the pathological appearances in relation to DDH only?

- ◯ Both hips - normal (1)
- ◯ Left hip appearances of DDH - Right hip normal (2)
- ◯ Left hip normal - Right hip appearances of DDH (3)
- ◯ Both hips have appearances of DDH (4)

Q73 Please indicate your confidence level for your decision regarding the presence of DDH. (1=no confidence, 10=full confidence)

	1	2	3	4	5	6	6	7	8	9	10
Confidence rating (1)											

Q75 How would you rate the technical quality (excluding exposure factors) for the above image (for suspected DDH only)?

- ◯ Perfect (1)
- ◯ Good (2)
- ◯ Moderate (3)
- ◯ Inadequate (4)

Page Break

End of Block: Image 17 (N50)

Start of Block: Image 18 (D8)

Q76 (**Image 18**) You are now asked to consider this image of a child with suspected Development Dysplasia of the Hip (DDH). You will asked to confirm whether this image is **normal** or **abnormal** (**for DDH only**) and indicate a **confidence rating** for your decision (0-not confident, 10-totally confident). You will also be invited to give **your opinion about the image quality**, this should consider ALL elements of image quality BUT NOT those which are exposure factor related (i.e. contrast, density, sharpness, noise).

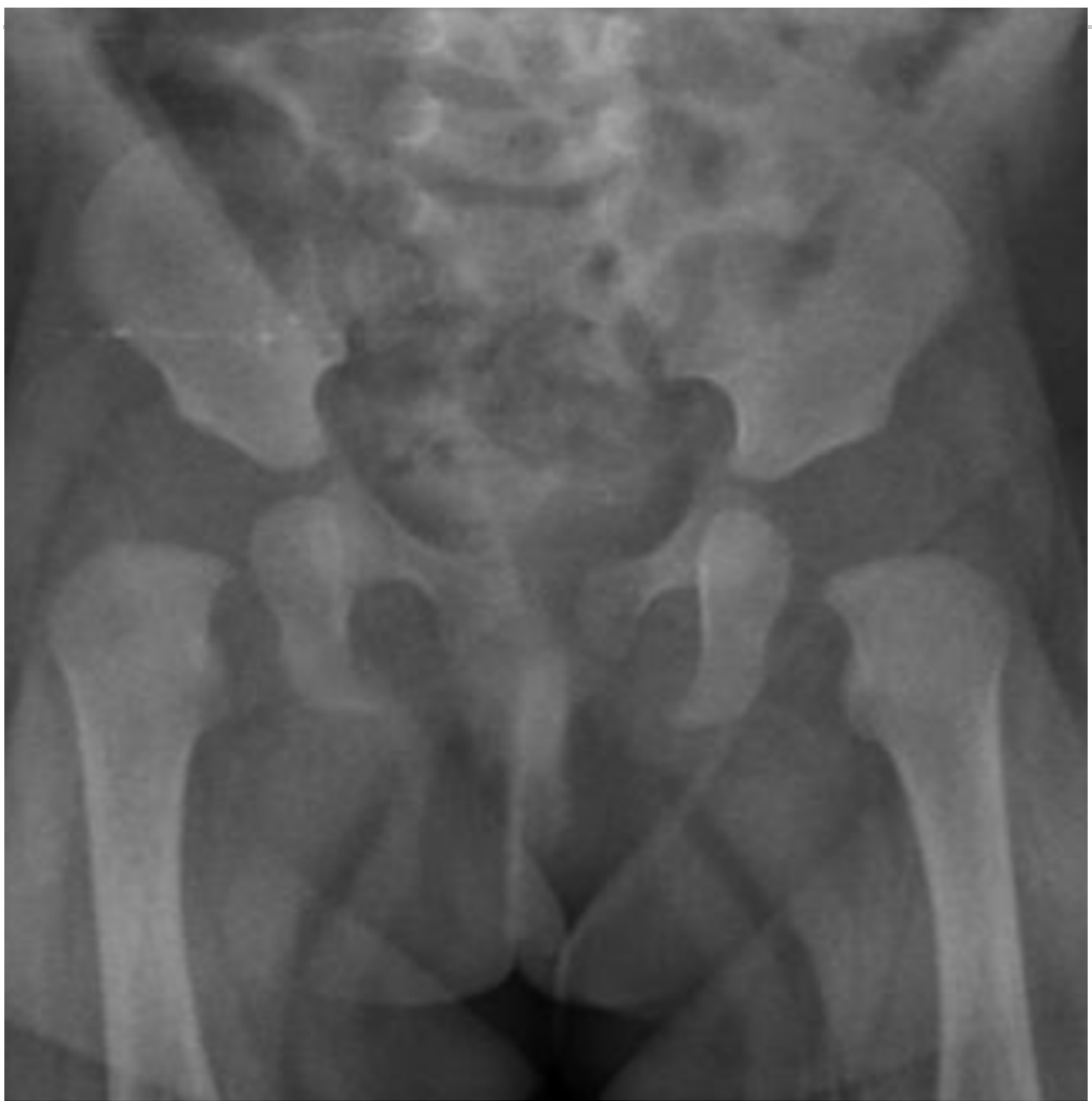

Q77 In relation to the image above, which best describes the pathological appearances in relation to DDH only?

- Both hips - normal (1)
- Left hip appearances of DDH - Right hip normal (2)
- Left hip normal - Right hip appearances of DDH (3)
- Both hips have appearances of DDH (4)

Q78 Please indicate your confidence level for your decision regarding the presence of DDH. (1=no confidence, 10=full confidence)

	1	2	3	4	5	6	6	7	8	9	10
Confidence rating (1)											

Q79 How would you rate the technical quality (excluding exposure factors) for the above image (for suspected DDH only)?

- Perfect (1)
- Good (2)
- Moderate (3)
- Inadequate (4)

Page Break

End of Block: Image 18 (D8)

Start of Block: Image 19 (D121)

Q80 (**Image 19**) You are now asked to consider this image of a child with suspected Development Dysplasia of the Hip (DDH). You will asked to confirm whether this image is **normal** or **abnormal** (**for DDH only**) and indicate a **confidence rating** for your decision (0-not confident, 10-totally confident). You will also be invited to give **your opinion about the image quality**, this should consider ALL elements of image quality BUT NOT those which are exposure factor related (i.e. contrast, density, sharpness, noise).

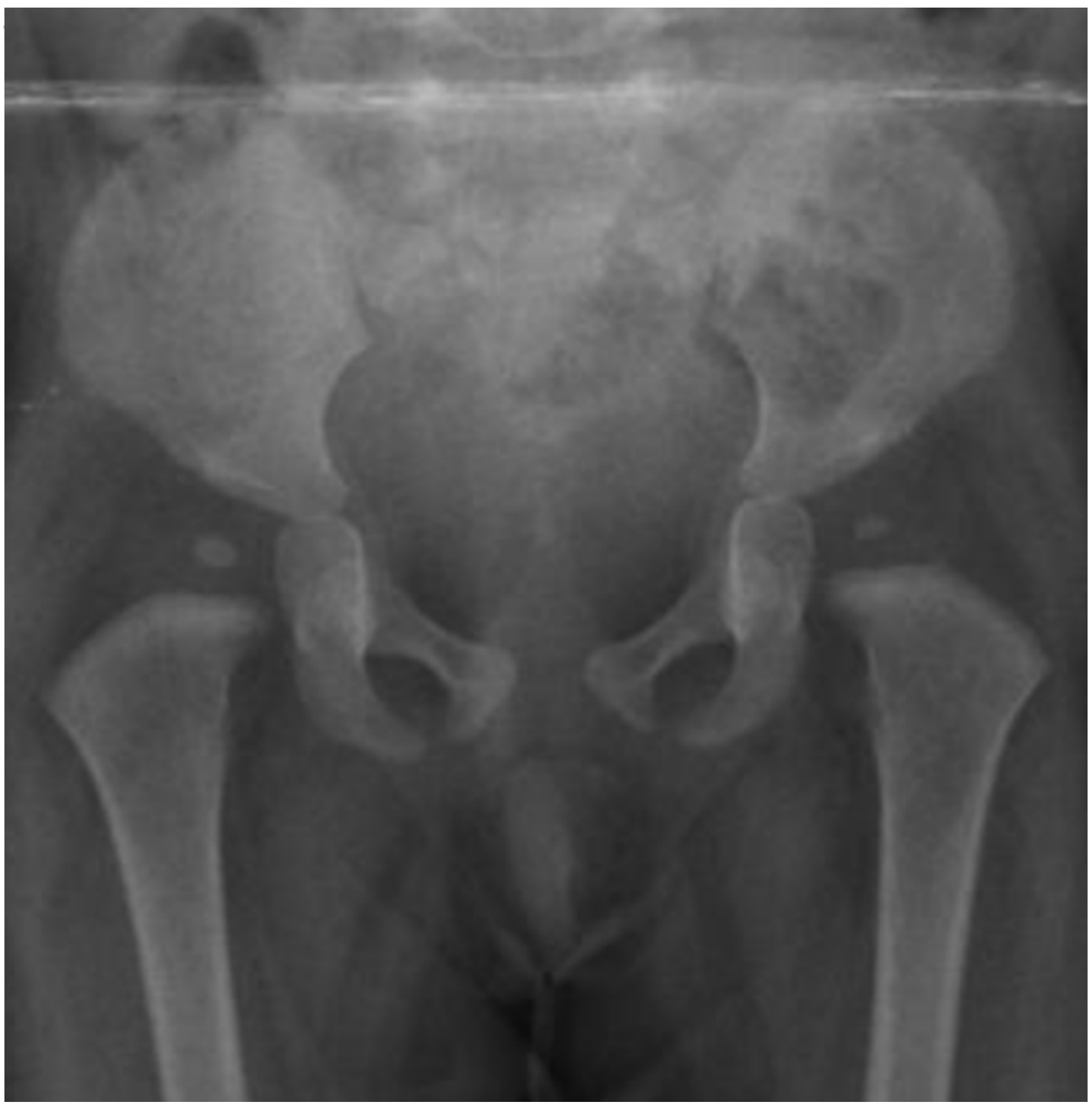

Q81 In relation to the image above, which best describes the pathological appearances in relation to DDH only?

- ◯ Both hips - normal (1)
- ◯ Left hip appearances of DDH - Right hip normal (2)
- ◯ Left hip normal - Right hip appearances of DDH (3)
- ◯ Both hips have appearances of DDH (4)

Q82 Please indicate your confidence level for your decision regarding the presence of DDH. (1=no confidence, 10=full confidence)

	1	2	3	4	5	6	6	7	8	9	10
Confidence rating (1)											

Q83 How would you rate the technical quality (excluding exposure factors) for the above image (for suspected DDH only)?

- ◯ Perfect (1)
- ◯ Good (2)
- ◯ Moderate (3)
- ◯ Inadequate (4)

Page Break

End of Block: Image 19 (D121)

Start of Block: Image 20 (D86)

Q84 (**Image 20**) You are now asked to consider this image of a child with suspected Development Dysplasia of the Hip (DDH). You will asked to confirm whether this image is **normal** or **abnormal** (**for DDH only**) and indicate a **confidence rating** for your decision (0-not confident, 10-totally confident). You will also be invited to give **your opinion about the image quality**, this should consider ALL elements of image quality BUT NOT those which are exposure factor related (i.e. contrast, density, sharpness, noise).

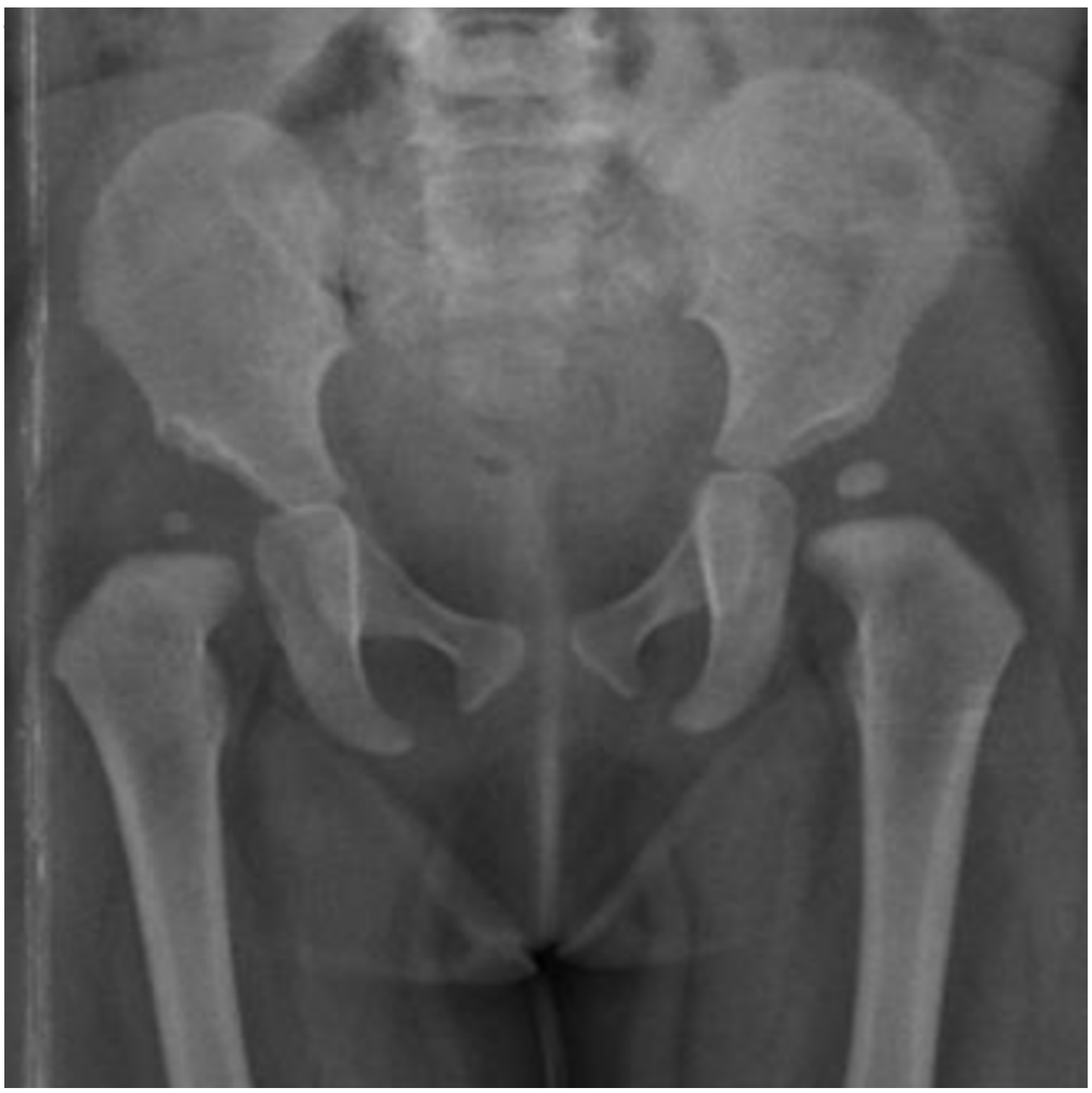

Q85 In relation to the image above, which best describes the pathological appearances in relation to DDH only?

- ◯ Both hips - normal (1)
- ◯ Left hip appearances of DDH - Right hip normal (2)
- ◯ Left hip normal - Right hip appearances of DDH (3)
- ◯ Both hips have appearances of DDH (4)

Q86 Please indicate your confidence level for your decision regarding the presence of DDH. (1=no confidence, 10=full confidence)

	1 2 3 4 5 6 6 7 8 9 10
Confidence rating (1)	

Q87 How would you rate the technical quality (excluding exposure factors) for the above image (for suspected DDH only)?

- ◯ Perfect (1)
- ◯ Good (2)
- ◯ Moderate (3)
- ◯ Inadequate (4)

Page Break

End of Block: Image 20 (D86)

Start of Block: Imager 21 (D107)

Q88 (**Image 21**) You are now asked to consider this image of a child with suspected Development Dysplasia of the Hip (DDH). You will asked to confirm whether this image is **normal** or **abnormal** (**for DDH only**) and indicate a **confidence rating** for your decision (0-not confident, 10-totally confident). You will also be invited to give **your opinion about the image quality**, this should consider ALL elements of image quality BUT NOT those which are exposure factor related (i.e. contrast, density, sharpness, noise).

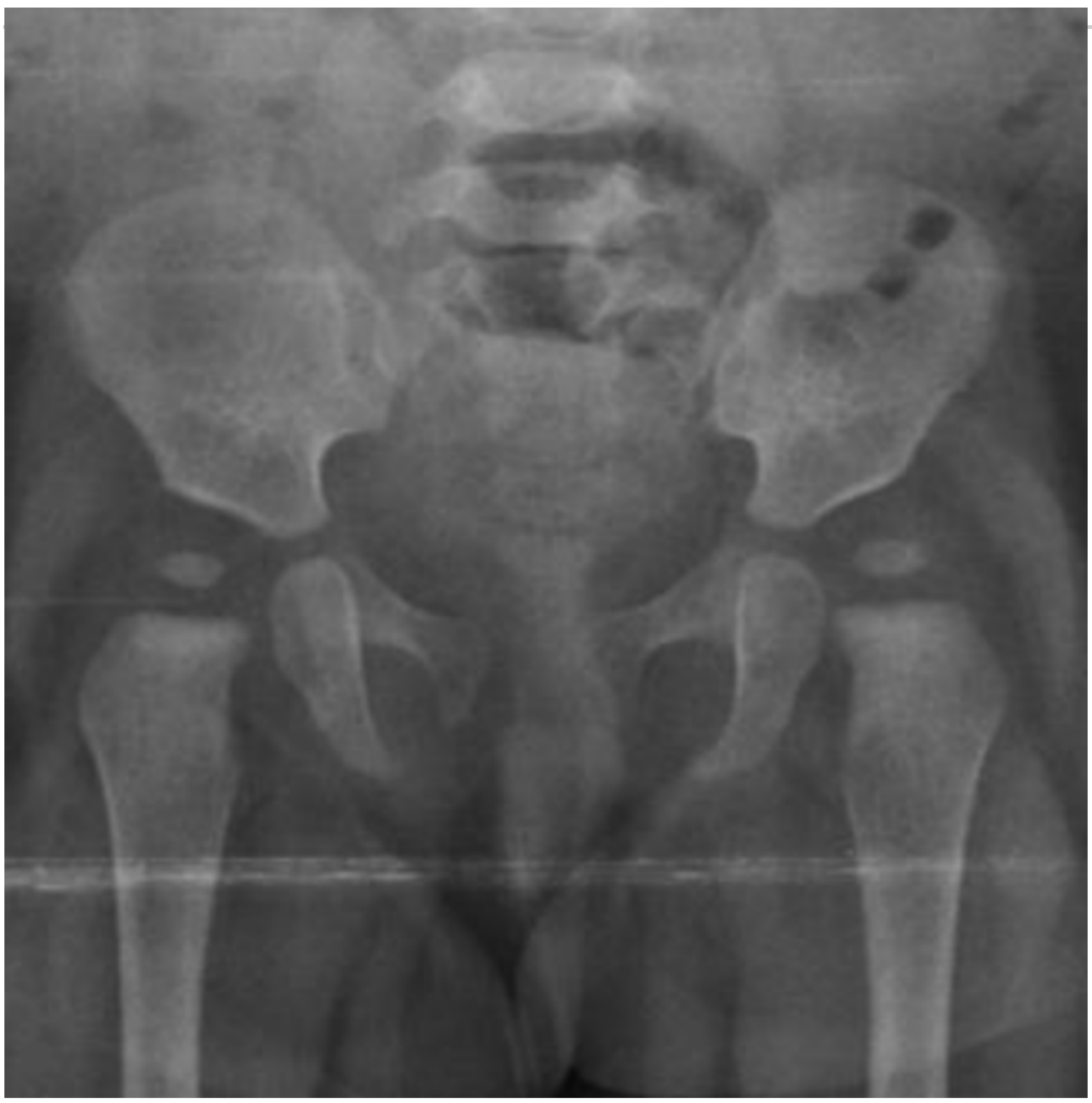

Q89 In relation to the image above, which best describes the pathological appearances in relation to DDH only?

- ◯ Both hips - normal (1)
- ◯ Left hip appearances of DDH - Right hip normal (2)
- ◯ Left hip normal - Right hip appearances of DDH (3)
- ◯ Both hips have appearances of DDH (4)

Q90 Please indicate your confidence level for your decision regarding the presence of DDH. (1=no confidence, 10=full confidence)

	1	2	3	4	5	6	6	7	8	9	10
Confidence rating (1)											

Q91 How would you rate the technical quality (excluding exposure factors) for the above image (for suspected DDH only)?

- ◯ Perfect (1)
- ◯ Good (2)
- ◯ Moderate (3)
- ◯ Inadequate (4)

Page Break

End of Block: Imager 21 (D107)

Start of Block: Image 22 (N314)

Q92 (**Image 22**) You are now asked to consider this image of a child with suspected Development Dysplasia of the Hip (DDH). You will asked to confirm whether this image is **normal** or **abnormal** (**for DDH only**) and indicate a **confidence rating** for your decision (0-not confident, 10-totally confident). You will also be invited to give **your opinion about the image quality**, this should consider ALL elements of image quality BUT NOT those which are exposure factor related (i.e. contrast, density, sharpness, noise).

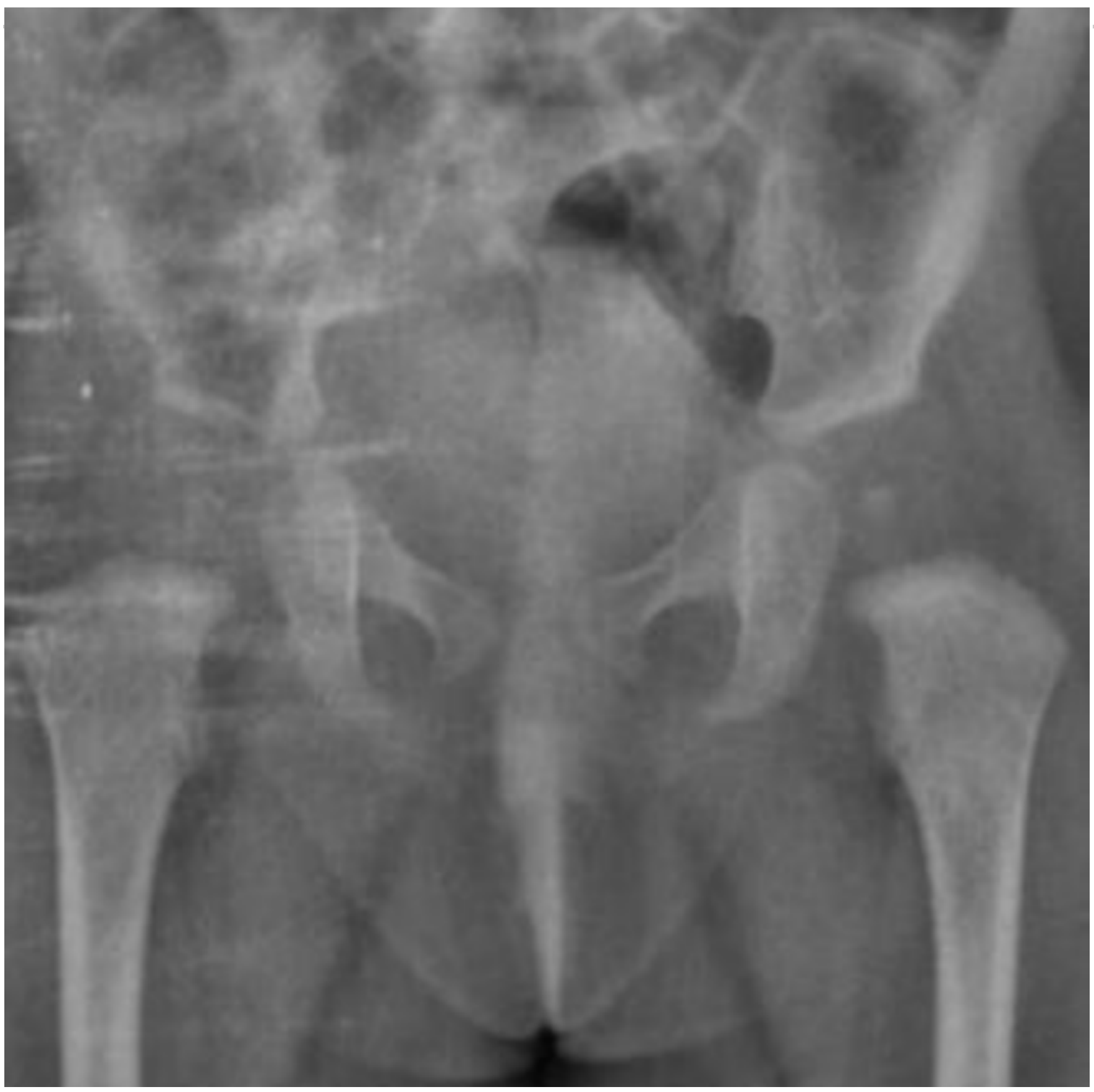

Q93 In relation to the image above, which best describes the pathological appearances in relation to DDH only?

- ◯ Both hips - normal (1)
- ◯ Left hip appearances of DDH - Right hip normal (2)
- ◯ Left hip normal - Right hip appearances of DDH (3)
- ◯ Both hips have appearances of DDH (4)

Q94 Please indicate your confidence level for your decision regarding the presence of DDH. (1=no confidence, 10=full confidence)

	1	2	3	4	5	6	6	7	8	9	10
Confidence rating (1)											

Q95 How would you rate the technical quality (excluding exposure factors) for the above image (for suspected DDH only)?

- ◯ Perfect (1)
- ◯ Good (2)
- ◯ Moderate (3)
- ◯ Inadequate (4)

Page Break

End of Block: Image 22 (N314)

Start of Block: Image 23 (N313)

Q96 (**Image 23**) You are now asked to consider this image of a child with suspected Development Dysplasia of the Hip (DDH). You will asked to confirm whether this image is **normal** or **abnormal** (**for DDH only**) and indicate a **confidence rating** for your decision (0-not confident, 10-totally confident). You will also be invited to give **your opinion about the image quality**, this should consider ALL elements of image quality BUT NOT those which are exposure factor related (i.e. contrast, density, sharpness, noise).

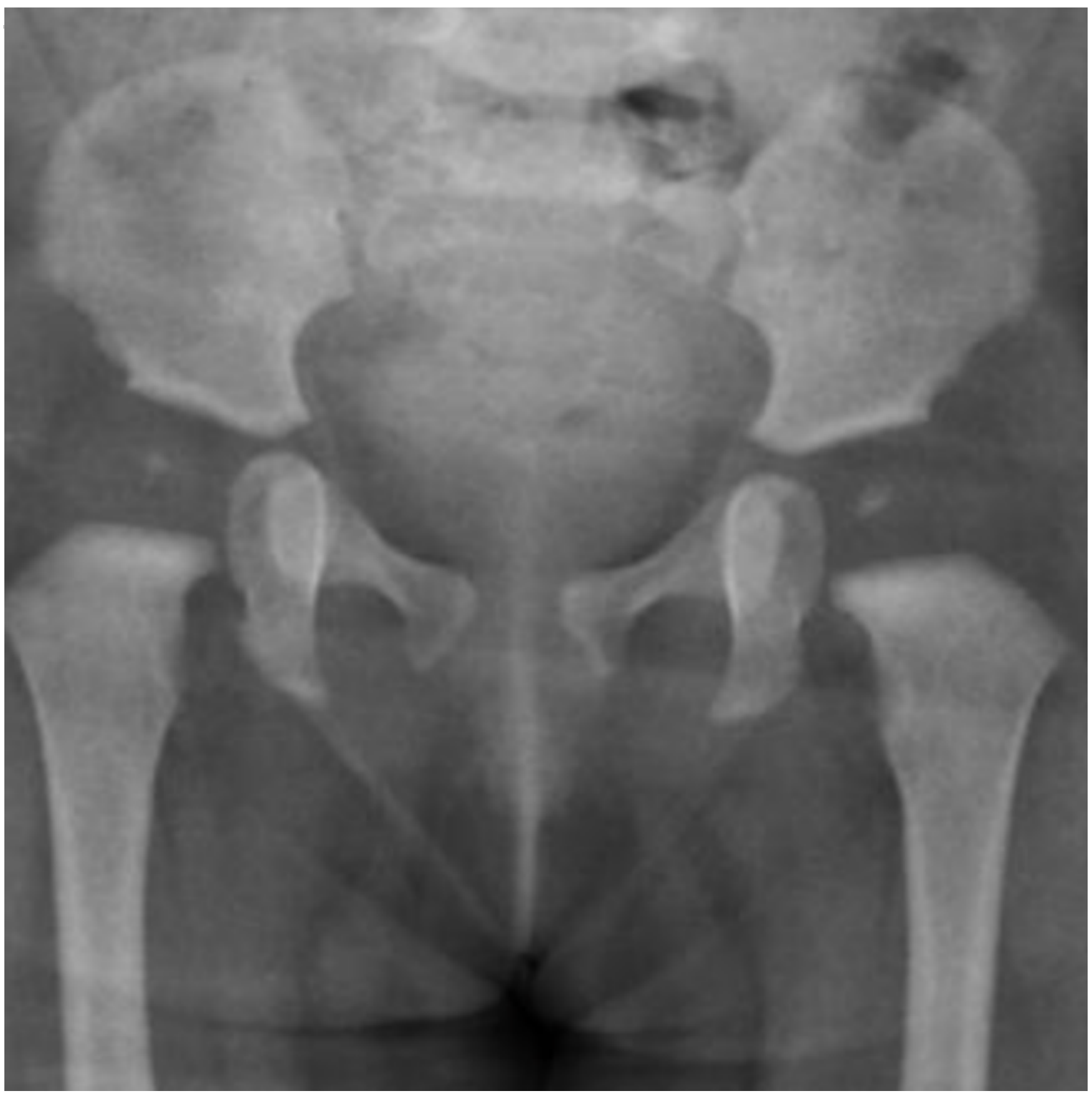

Q97 In relation to the image above, which best describes the pathological appearances in relation to DDH only?

- ◯ Both hips - normal (1)
- ◯ Left hip appearances of DDH - Right hip normal (2)
- ◯ Left hip normal - Right hip appearances of DDH (3)
- ◯ Both hips have appearances of DDH (4)

Q98 Please indicate your confidence level for your decision regarding the presence of DDH. (1=no confidence, 10=full confidence)

	1	2	3	4	5	6	6	7	8	9	10
Confidence rating (1)											

Q99 How would you rate the technical quality (excluding exposure factors) for the above image (for suspected DDH only)?

- ◯ Perfect (1)
- ◯ Good (2)
- ◯ Moderate (3)
- ◯ Inadequate (4)

Page Break

End of Block: Image 23 (N313)

Start of Block: Image 24 (N268)

Q100 (**Image 24**) You are now asked to consider this image of a child with suspected Development Dysplasia of the Hip (DDH). You will asked to confirm whether this image is **normal** or **abnormal** (**for DDH only**) and indicate a **confidence rating** for your decision (0-not confident, 10-totally confident). You will also be invited to give **your opinion about the image quality**, this should consider ALL elements of image quality BUT NOT those which are exposure factor related (i.e. contrast, density, sharpness, noise).

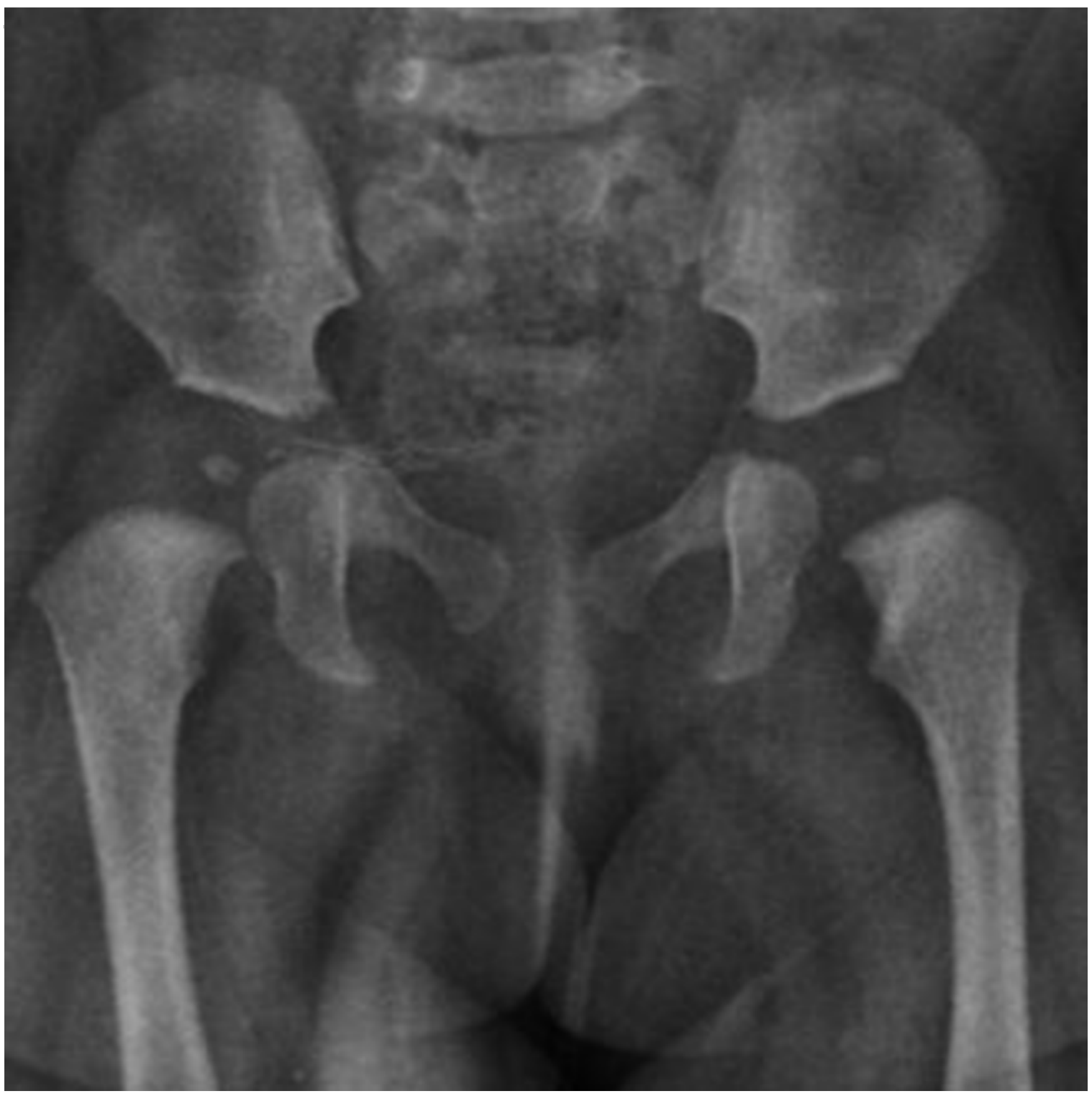

Q101 In relation to the image above, which best describes the pathological appearances in relation to DDH only?

- ◯ Both hips - normal (1)
- ◯ Left hip appearances of DDH - Right hip normal (2)
- ◯ Left hip normal - Right hip appearances of DDH (3)
- ◯ Both hips have appearances of DDH (4)

Q102 Please indicate your confidence level for your decision regarding the presence of DDH. (1=no confidence, 10=full confidence)

	1	2	3	4	5	6	6	7	8	9	10
Confidence rating (1)											

Q103 How would you rate the technical quality (excluding exposure factors) for the above image (for suspected DDH only)?

- ◯ Perfect (1)
- ◯ Good (2)
- ◯ Moderate (3)
- ◯ Inadequate (4)

Page Break

End of Block: Image 24 (N268)

Start of Block: Image 25 (N304)

Q104 (**Image 25**) You are now asked to consider this image of a child with suspected Development Dysplasia of the Hip (DDH). You will asked to confirm whether this image is **normal** or **abnormal** (**for DDH only**) and indicate a **confidence rating** for your decision (0-not confident, 10-totally confident). You will also be invited to give **your opinion about the image quality**, this should consider ALL elements of image quality BUT NOT those which are exposure factor related (i.e. contrast, density, sharpness, noise).

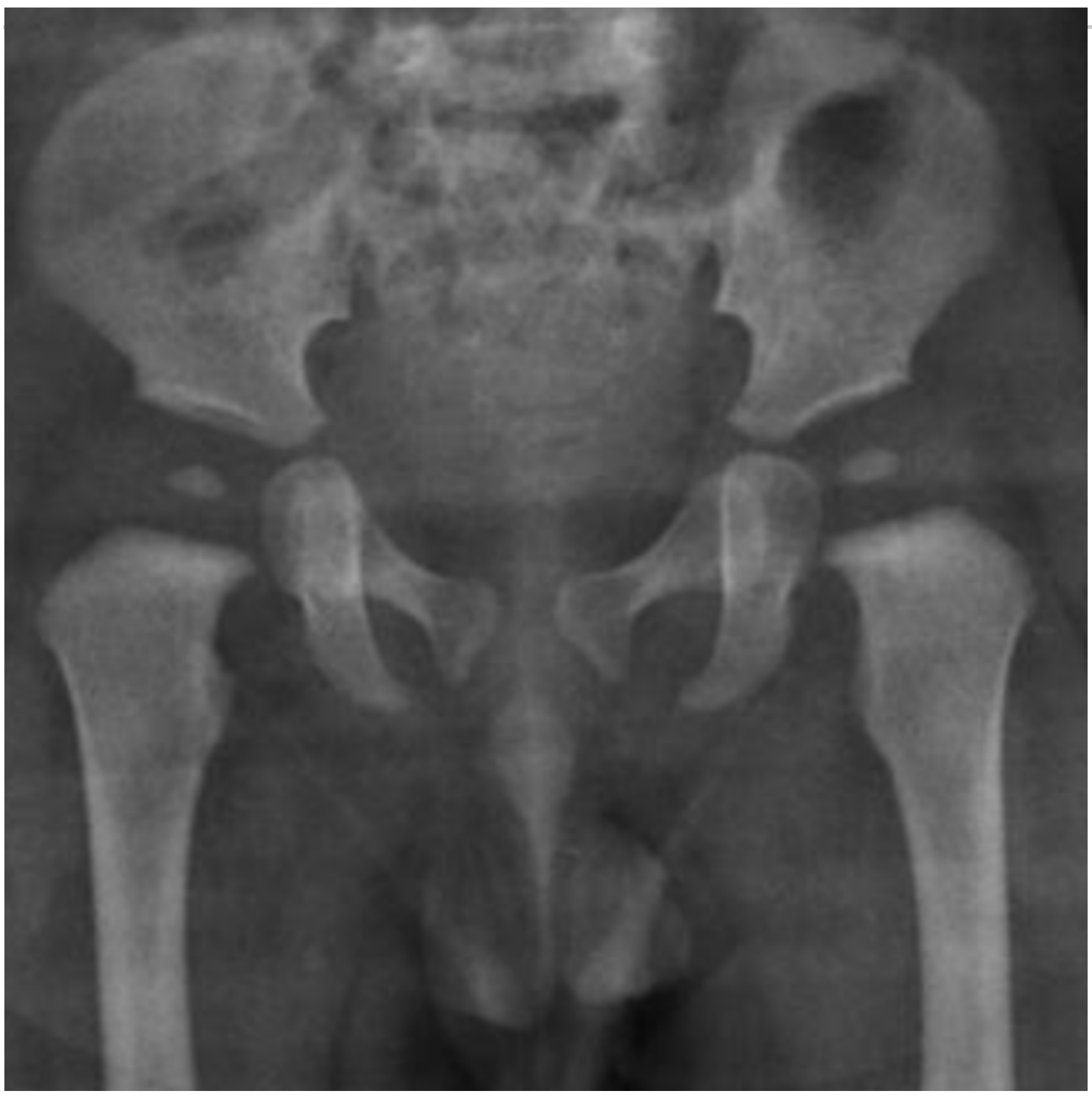

Q105 In relation to the image above, which best describes the pathological appearances in relation to DDH only?

○ Both hips - normal (1)

○ Left hip appearances of DDH - Right hip normal (2)

○ Left hip normal - Right hip appearances of DDH (3)

○ Both hips have appearances of DDH (4)

Q106 Please indicate your confidence level for your decision regarding the presence of DDH. (1=no confidence, 10=full confidence)

	1 2 3 4 5 6 6 7 8 9 10
Confidence rating (1)	

Q107 How would you rate the technical quality (excluding exposure factors) for the above image (for suspected DDH only)?

○ Perfect (1)

○ Good (2)

○ Moderate (3)

○ Inadequate (4)

Page Break
